# Analyzing the regulatory framework gaps for gas distribution networks with decreasing natural gas demand in Germany

**DOI:** 10.1016/j.heliyon.2024.e40800

**Published:** 2024-12-05

**Authors:** Stella Oberle, Till Gnann, Louis Wayas, Martin Wietschel

**Affiliations:** a Fraunhofer Institution for Energy Infrastructures and Geothermal Systems IEG, Breslauer Str. 48, 76139 Karlsruhe, Germany; b Fraunhofer Institute for Systems and Innovation Research ISI, Breslauer Str. 48, 76139 Karlsruhe, Germany; c DVGW Research Centre at the Engler-Bunte Institute of the KIT, Engler-Bunte-Ring 1-9, 76131, Karlsruhe, Germany

**Keywords:** Gas distribution network, Network regulation, Revenue cap, Decommissioning, Network charges

## Abstract

German energy system studies, investigating the energy transition pathways to the set climate targets, depict a significant decrease in gas demand. This leads to a discussion about the long-term need of gas distribution networks. The discussion intensified with the war in Ukraine and the subsequent energy price crisis. The German regulatory agency responded to these developments with adjusting the regulatory framework to the current challenges. Up to now, the depreciation period of network components varies between 45 and 65 years, and consequently the monetary capital is tied up for a long time, lowering the flexibility of network operators to react to current challenges. Therefore, the German regulatory agency allows the shortening of depreciation periods of new gas network assets. Nonetheless, it is still unclear how to deal with existing assets at risk of becoming stranded assets and how to regulate the decommissioning of gas networks. Therefore, this paper addresses the research question: “What effect do different regulations for decommissioning of gas distribution networks have on operators and users?“. To answer the question the model MERLIN is applied, which integrates the current and future regulation options into long-term investment decision analysis. The results show a need to shorten the depreciation period of existing assets to avoid stranded assets. Further, partial decommissioning with including the decommission costs in the regulatory framework and hence finance it through the network users, is the most economical attractive option for network operators and users.

## Highlights

• Long-term investment analysis including current and future regulatory conditions.
• High risk of significant future stranded assets based on current network assets.
• Including partial decommissioning costs in revenue cap most attractive option.
• Financing of full dismantling costs with provisions leads to breach of regulation.
• Efficiency factor has high influence on economic feasibility of network operation.

## Abbreviations:

ARegV: Incentive Regulation Act (Anreizregulierungsverordnung)
BMWK: Federal Ministry for Economic Affairs and Climate Actions
CAPEX: Capital expenditure
CCD_t_: Capital cost deduction in year t
CC_t_: Capital costs in year t
CHP: Combined Heat and Power
DP_t_: Calculated depreciation in year t
D_t_: Distribution factor in year t
d_t_: Gas demand in year t
DVGW: German Technical and Scientific Association for Gas and Water
Eff: Efficiency Factor
E_t_: Expenses in year t
EU: European Union
GHG: Greenhouse Gas
I_0_: Investment at the beginning
k: Thousand
KIT: Karlsruhe Institute of Technology
L_n_: Liquidity revenue at the end of lifetime
Mio: Million
NC_t_: Network charges in year t
NEG: Network ownership company (Netzeigentumsgesellschaft) Rheinstetten GmbH & Co. KG
NPV: Net present value
OPEX: Operational expenditure
RC_t_: Revenue cap in year t
RoD_t_: Return on dept in year t
RoE_t_: Calculated return on equity in year t
R_t_: Revenue in year t
SWE: Municipal utility Esslingen
SWK: Municipal utility Karlsruhe
SWKN: Municipal utility Karlsruhe network services GmbH
TN: GHG neutral
TNC_t_: Total network costs in year t
TOTEX: Total expenditures
TT_t_: Calculated trade tax in year t

## 1 Introduction

With the Green Deal, the EU commits to reach net zero greenhouse gas (GHG) emissions by 2050 [1] and the German Climate Change Act states the aim to achieve climate neutrality already until 2045 [2]. These targets require a variety of GHG reduction measures, such as energy efficiency measures to reduce energy demand in different demand sectors and the integration of renewable energy along with the phase out of fossil fuels. Russia's invasion of Ukraine and the resulting energy crisis intensifies the discussion to phase out natural gas in Germany [3].

These developments lead to uncertainties of political decision making, which increases the risks of investment decisions, especially for the gas network [4]. This affirms the decision in some countries, such as the UK, to phase out the installation of gas boilers in houses by 2035 [5] and the discussion in Germany to allow only new heating systems with a share of 65 % renewable energy from 2024 onwards [6]. Generally [4], see the future of the gas network as one big challenge itself, because it is one of the most important assets in many countries and the demand development in various studies, such as [[7], [8], [9], [10]], show a decrease in gas demand, hence a decreasing need of gas networks. The studies see these developments despite the existing decarbonization options for gas networks, such as synthetic methane, biogas and hydrogen [4]. A strong gas demand decrease with constant network length and cost results in an extreme increase in network charges, since the network costs are distributed among fewer network users [11,12].

Further, the regulatory framework for gas distribution network operators stipulates the depreciation period of the various network assets, e.g. 45–65 years for gas pipelines [13]. These long depreciation periods bind the monetary capital for a long time, lowering the flexibility of network operators to react to current challenges. Taking the target of climate neutrality until 2045 into consideration, investments into the gas network from the last decades are in danger of becoming stranded investments. This uncertainty of future gas demand and the restrictive regulatory framework establishes a challenging environment for gas network operators to make future-oriented investment decisions.

In November 2022, the German federal regulatory authority decided to allow shorter depreciation periods for new assets activated from 2023 onwards to address this issue [3]. This adjustment allows the network operators to decide to limit its depreciation period until 2045 for every new asset. Hence, the depreciation period of pipelines activated in 2023 is allowed to be only 22 years [3]. While the majority of stakeholders welcome this decision, for most of them it is still not sufficient. The stakeholders claim that the depreciation period of existing assets should be allowed to be shortened as well. Further, the decommissioning of gas pipelines is not part of the regulation and should be addressed in future [3].

For this reason, we investigate the different regulatory options to address the limited use of the gas distribution network and its decommission in this paper. With our analysis we contribute to the scientific community by answering the question:

“What effect do different regulations for decommissioning of gas distribution networks have on operators and users?”

To answer this question, we focus on the German regulatory framework, because of the high amount of gas distribution network operators affected by the energy transition and the important role of natural gas in Germany. Further, the time frame of this investigation is between 2018 until2050,^1^ including the base year 2015 for the third regulation period.

In the following, we first provide an overview of the current literature investigating the future of gas distribution networks and its regulatory framework. Afterwards, the regulatory frameworks for gas networks in the EU and in Germany are explained. The third section describes the method and model used including its validation. Thereafter, the data needed and the scenarios investigated are depicted and afterwards the results shown. After discussing the results, conclusions with recommendations for adaptations and gaps in the regulation are presented.

## 2 The current situation of gas networks

In this section, first the current literature, analyzing the future role of gas distribution networks, is shown by starting with studies from a nationwide perspective to studies accessing typical networks until investigations based on real network data. After identifying the research gaps in the current literature, an insight into the network regulation in different countries is given, followed by a detailed explanation of the German network regulation. In the end, different regulation options for considering the decommission of gas distribution networks are pointed out.

### 2.1 Literature on the future role of gas distribution networks

There are several studies investigating the future role of natural gas or synthetic methane (in the following only gas) in the energy system as well as analyses on gas distribution networks. Studies with a nationwide perspective investigating the transformation of the German energy system and the measures needed to achieve climate neutrality until 2045 depict a strong gas demand decrease, especially in the demand sectors mainly supplied by the gas distribution network [[7], [8], [9], [10]]. While [9] takes a microeconomic perspective on the German energy system [7,8,10], conduct macroeconomic analyses [8]. points out that gas distribution networks will no longer be sufficiently utilized and hence parts of the networks need to be decommissioned. However, detailed modelling of gas distribution networks and its decommission is not carried out. A quantitative and qualitative analysis of the gas and hydrogen network is included in Ref. [7], but only [10] models gas distribution networks and quantifies its decommission.

Studies focusing on the distribution network operators, such as [14], underline the key role of distribution network operators in the energy transition and point out that the current regulatory framework is rather preventing the transition needed [15]. include different regulatory adjustments, for example shortening the depreciation period of assets or considering a risk surcharge for higher equity interest rates, while simulating gas networks based on typical network schemes. However, decommissioning of the gas network is not taken into account [15]. In contrast [16], assesses the decommission of gas networks in detail by investigating energy systems with and without a gas infrastructure [11,17]. base their analysis on the decommission cost assumptions of [16]. [17] shows different gas infrastructure transition pathways towards climate neutrality in France and Germany, including the decommissioning of some parts of gas distribution networks [11]. analyze model networks with detailed gas network modelling and determination of decommission costs. Further, the study points out a need to restructure the network refinancing and to adjust the regulatory framework [11].

The above-mentioned studies include gas network simulation based on typical networks or model networks and are overall system optimizations. In contrast [18], optimize the gas distribution network of one region with half a million inhabitants. The gas distribution networks of different counties in Germany are optimized by Ref. [19]. The analysis includes the decommissioning of parts of the gas network and the cost assumptions are based as well on [16]. [19] points out the need of new standards in network finance and planning in the light of gas network decommission [20]. goes even further and claims that the heat supply of the building sector is possible without a gas distribution network and even favorable. Further [21], conducts an analysis on the role of gas distribution networks in a decarbonized energy system with Ireland as a use case. They use a financial model including an integrated energy system model approach [21].

All the above-mentioned approaches conduct a detailed assessment of gas distribution networks considering its decommissioning and having partly a high regional resolution. Nonetheless, none of them consider the regulatory framework. To the best of the authors' knowledge, only [[22], [23], [24]] investigate the interdependencies between investment decisions of gas network operators and network users within the current regulatory framework [22]. starts the analysis with an assessment of the network length and the number of network users by power law. Further, the development of network charges is analyzed by considering different strategy pathways and a simplified version of the revenue cap. While [22] focuses on the gas network [23], conduct an agent-based simulation of the electricity and gas network in combination with an optimization of building renovations. This approach is used to investigate the interactions between building refurbishment measures on the gas and electricity demand and the effect from the network operator's investment decisions on the network charges. It includes a simplified revenue cap based on yearly network costs [23]. Based on [[22], [23], [24]] analyses the interactions between investment decisions of electricity and gas network operators and building owners with a mixed integer linear optimization model. All three publications do not consider the decommissioning of gas networks.

This status quo of the literature on the future role of gas distribution networks in a decarbonized energy system shows that the majority of studies depict a necessity to decommission parts of gas distribution networks, however on different levels of detail. Most of them do not consider the regulatory framework, while a small amount shows its influence and challenges. Only [[22], [23], [24]] include a simplified revenue cap with annual network costs, focusing on assessing the interplay between electricity and gas network operators and building owners. This leads to research gaps in assessing the regional decommissioning costs, their effect on network operators and network users as well as the influence of the regulatory framework including its time dependencies, such as resulting time delays from defined regulation periods. For this reason, in the following an assessment of different decommission strategies with different regulatory options is conducted. In the following, the different regulatory frameworks will be explained.

### 2.2 Gas network regulation

Gas networks are natural monopolies as it is economically questionable to operate pipelines from different operators in parallel [25]. This situation, however, has the danger of being misused by the network operators, for example by asking for disproportionately high network charges. Consequently, network operators are strictly regulated [25]. The regulation methods are differentiated in cost or incentive regulations [25,26]. For the cost regulation the instruments of rate-of-return regulation and of cost-plus regulation are applied. By applying the rate-of-return regulation the regulatory authority provides a return to the network operator based on a certain rate of return on capital [26]. In the cost-plus regulation the network operator is allowed to have a proportional profit margin on the network costs [26]. In contrast, in the incentive regulation revenues and network costs are decoupled for a certain time period (regulation period), so that a reduction in costs during this period leads to higher revenues [25]. The instruments for incentive regulation are price cap, revenue cap and yardstick competition [26]. The price cap is set by the regulatory authority before the start of the regulation period. During the regulation period it is adjusted annually by inflation (change in the consumer price index) and a target set by the regulatory authority to increase productivity. For the revenue cap the regulatory authority limits the revenues generated. Both of the earlier mentioned incentive regulation instruments can be combined with yardstick competition. Therefore, an efficiency comparison between structurally similar network operators is carried out by the regulatory authority, determining the efficiency factor for the revenue cap instrument or the productivity target for the price cap [26].

The regulatory framework for gas distribution network varies from country to country. Table 1 provides an overview of the gas distribution network regulation in selected European countries. The majority of the selected European countries have an incentive-based regulation of gas distribution networks with the revenue cap instrument and a regulation period of up to five years.

**Table 1:** Gas distribution network in different countries and its regulation (status 2021) [27,28].

Country	Network operator	Network length	Regulation	Regulation period
Germany	665	554,500 km	Incentive-based with revenue cap	5 years
Italy	∼194	∼226,000 km	Cost plus for capital costs, price cap for operating costs	6 years
Austria	21	44,000 km	Incentive-based with revenue cap	5 years
Finland	19	∼2000 km	Incentive-based with revenue cap	4 years
Netherlands	7	125,000 km	Incentive-based with price cap	3–5 years
Ireland	1	∼11,913 km	Incentive-based with revenue cap	5 years

Also a comparison of the regulatory frameworks in Europe, conducted by Ref. [28], concludes that most countries use incentive regulation with a revenue cap, while cost-plus regulation is only used by a few countries and is therefore an exception. In the following, the German regulatory framework is analyzed due to the important role of natural gas in Germany to date, the very ambitious German GHG reduction targets and the countries drive to quickly reduce its dependence on gas due to the Ukraine conflict.

#### 2.2.1 Incentive regulation with revenue cap in Germany

Before a regulation period starts, a cost evaluation is performed by the regulatory authority based on the base year [25,29]. The base year is the third year of the previous regulation period and hence for the fourth regulation period (2023–2027) the base year is 2020. In the last year of each regulation period the efficiency comparison is conducted by the regulatory authority for the next regulation period [25].

The costs evaluated are operational expenditures (OPEX), mainly cost of materials or personnel costs, and capital expenditures (CAPEX), such as interest on equity and depreciation [25,30]. Together these costs are the total expenditures (TOTEX), which result in the network costs after subtracting the cost decreasing revenues, other income and releases of construction cost subsidies [25]. Based on these network costs, the regulatory authority determines the revenue cap by applying the following regulation formula (ARegV annex 1 to §7) [31]:

[Eq. 1] $${RC}_{t}={CS}_{pnc,t}+\left({CS}_{tnc,t}+\left(1-D_{t}\right)\cdot {CS}_{c,t}+\frac{B_{0}}{T}\;\right)\cdot \left(\;\frac{{CPI}_{t}}{{CPI}_{0}}-{PF}_{t}\right)+{CCM}_{t}+Q_{t}+\left({VC}_{t}-{VC}_{0}\right)+S_{t\;}$$

With:

${RC}_{t}$ Revenue cap in € in year t

${CS}_{pnc,t}$ Permanent non-controllable cost share in € in year t

${CS}_{tnc,t}$ Temporary non-controllable cost share in € in year t

$D_{t}$ Distribution factor for the reduction of inefficiencies in year t

${CS}_{c,t}$ Controllable cost share in € in year t

$B_{0}$ Efficiency bonus in € in base year

T Time period of five years

${CPI}_{t}$ Consumer price index in year t

${CPI}_{0}$ Consumer price index in base year

${PF}_{t}$ General sectoral productivity factor in year t

${CCM}_{t}$ Capital cost mark-up in € in year t

$Q_{t}$ Quality element in € in year t

${VC}_{t}$ Volatile cost element in € in year t

${VC}_{0}$ Volatile cost element in € in base year

$S_{t\;}$ Sum of surcharge and deductions of regulation account in € in year t according to § 5 para. 3 ARegV

The regulatory network costs are split into controllable and non-controllable cost elements. Permanent non-controllable costs^2^ are part of the regulation formula (as shown in equation (1)) but are not included in the efficiency comparison [29] as these costs are considered efficient and necessary for a certain social standard [25]. These costs are ground on the base year, however larger changes can be included during the regulation period to adjust the revenue cap.

The other cost elements are controllable costs (${CS}_{c,t},{CS}_{tnc,t}$) and underly the efficiency comparison [25]. The inefficiencies are the so-called controllable cost shares (${CS}_{c,t}$) that need to be reduced during the regulation period. Therefore, the distribution factor ($D_{t}$) is distributing these cost shares over the five years of the regulation period. To calculate the controllable cost share the permanent non-controllable costs (${CS}_{pnc,0}$), the temporarily non-controllable costs (${CS}_{tnc,t}$) and the capital cost deduction (${CCD}_{t}$) is subtracted from total network costs (${TNC}_{t}$), as equation (2) shows [25,31].

[Eq. 2] $${CS}_{c,t}={TNC}_{t}-{CS}_{pnc,0}-{CCD}_{t}-{CS}_{tnc,t}$$

Temporarily non-controllable costs (${CS}_{tnc,t}$) are the efficient part of the controllable cost and consequently do not need to be reduced during the regulation period [25]. These cost shares are calculated according to equation (3) [31]. The efficiency factor (Eff) is the result of the efficiency comparisons and its range is between 60 % and 100 %, meaning 60 % is the least efficient network operator and 100 % the most efficient [29]. Hence, a network operator with an efficiency factor of 70 % has 30 % inefficient controllable costs and 70 % efficient controllable costs (temporarily non-controllable costs).

[Eq. 3] $${CS}_{tnc,t}=\left({TNC}_{t}-{CS}_{pnc,0}-{CCD}_{t}\right)\cdot Eff$$

With the capital cost deduction (${CCD}_{t}$) the impairment of the network components is determined and taken into account in the controllable cost (${CS}_{c,t}$, ${CS}_{tnc,t}$) [25]. It is determined before the regulation period and is the difference between the capital costs in the base year (${CC}_{0}$) and the capital costs in the different years of the regulation period (${CC}_{t}$), as shown in equation (4) [29,31]. The corresponding capital costs are the sum of calculated depreciation (${DP}_{t}$), calculated return on equity (${RoE}_{t}$), calculated trade tax (${TT}_{t}$) and return on debt (${RoD}_{t}$) (equation (5)).

[Eq. 4] $${CCD}_{t}={CC}_{0}-{CC}_{t}$$

[Eq. 5] $${CC}_{t}={DP}_{t}+{RoE}_{t}+{TT}_{t}+{RoD}_{t}$$

Other elements of the regulation formula are very briefly explained in Appendix B. More detailed explanations can be found in Refs. [13,25,31,32].

### 2.3 Decommissioning of gas networks

**Fig. 1:**
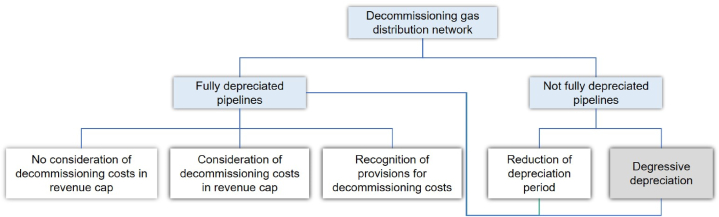
Regulation options for considering decommissioning of gas distribution networks based on [34].

First projects with a complete dismantling of the gas distribution network are carried out in Switzerland in Zurich and St. Gallen [33,34]. As in Germany, Switzerland does not yet regulate the decommissioning of gas networks. Therefore [34], compiled different regulation options for the decommissioning of gas networks, which are illustrated in Fig. 1. Decommissioning can take place before or after an asset's depreciation period is over, meaning before or after the investment is refinanced by network charges. To prevent the decommissioning of assets with remaining depreciation period and hence avoiding additional costs due to decommission, the depreciation process can be accelerated. Therefore, the depreciation period can be shortened [34]. This is already practiced in New Zealand and the UK [35]. In November 2022, Germany also decided to allow the optional reduction of the depreciation period of new assets activated in 2023 or later [3].

Alternatively, the depreciation process can be transformed from linear to degressive depreciation. In a degressive depreciation, the depreciation rate is calculated annually from the residual value, in contrast to the linear depreciation, in which the depreciation rate is constant [34]. This results in a decreasing depreciation rate over the course of the lifetime and consequently, to a significantly higher depreciation at the beginning of the lifetime than towards the end [34]. Since the German regulatory authority already decided on the shorter depreciation period for new assets, in this paper it is assumed that this will also apply for existing assets.

If the asset is already fully depreciated or the depreciation period shortened accordingly, three options can be applied to deal with the costs of decommissioning [34]. The first option is that costs are not considered in the revenue cap, meaning that the network operators have to cover the costs of decommissioning by themselves. Secondly, decommission costs could be allowed to be considered in the revenue cap, which leads to the network user covering the decommissioning costs. Lastly, the regulatory authority allows the network operator to arrange provisions for the decommissioning [34]. evaluated the different regulation options for Switzerland, which cannot be directly considered for Germany, because it applies a cost-plus regulation. Nonetheless, the different regulation options can be helpful to fill the regulation gap in Germany. Therefore, in the following these options are assessed in detail.

## 3 Methodology and data assumptions

In the following section the model implemented is introduced and an overview of the data assumptions needed for the assessment in this paper are outlined.

### 3.1 Model MERLIN

To assess the before mentioned regulation options including decommissioning of gas distribution networks this paper combines a detailed consideration of the regulatory framework with the evaluation of investment options using the net present value method and links it to long-term gas demand scenarios. This is performed by using the Python-based model MERLIN (Municipal energy infrastructure investment analysis under regulation). The model takes the microeconomic perspective of one network operator for a long-term simulation from 2018 to 2050 (including the base year 2015).

**Fig. 2:**
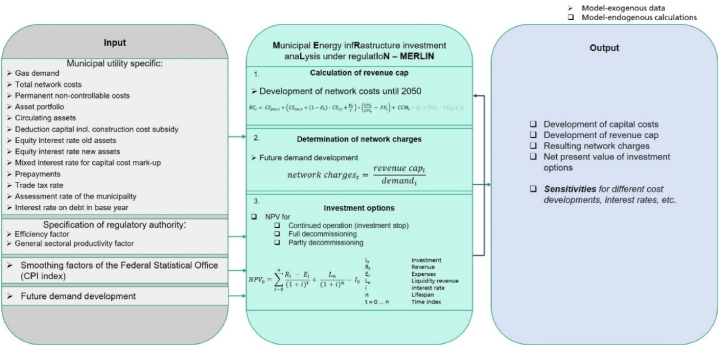
Overview of model MERLIN, its input data and model output (own illustration).

An overview of the models' input data needed, its different modules and its resulting output is shown in Fig. 2. The input data in the left panel are mainly provided by network operators and to a smaller extent by the regulatory authority and the federal statistical office. This will further be explained in Section 3.2. In the center, the methodology of MERLIN is shown, which starts with the implemented regulation formula (see equation (1)) and is further explained in Section 3.1.1. After calculating the revenue cap, the annual average network charges for the network investigated are determined based on the revenue cap calculated and the model-exogenous demand development (section 3.1.2). The development of the network charges indicates the effect on the network user. In the end, the investment options are evaluated by calculating their net present values (section 3.1.3), providing an insight into the effect on the network operator. The different investment options change the asset base for calculating the revenue cap, so that depending on the investment options different revenue caps and network charges are derived. The results of the model and its sensitivities (Fig. 2 right panel) are explained in section 4.

#### 3.1.1 Implementation of regulatory framework (module 1)

As a first step, the revenue cap before the start of a regulation period is determined by using the shortened regulation formula in equation (6). The capital cost mark-up, the volatile costs and the balance of the regulation account are parts of the adjustment of the revenue cap during the regulation period and explained afterwards. In this paper, we are solely considering the gas distribution network, hence the quality element is not considered as well.

[Eq. 6] $${RC}_{t}={CS}_{pnc,t}+\left({CS}_{tnc,t}+\left(1-D_{t}\right)\cdot {CS}_{c,t}+\frac{B_{0}}{T}\;\right)\cdot \left(\;\frac{{CPI}_{t}}{{CPI}_{0}}-{PF}_{t}\right)$$

With:

${RC}_{t}$ Revenue cap in € in year t

${CS}_{pnc,t}$ Permanent non-controllable cost share in € in year t

${CS}_{tnc,t}$ Temporary non-controllable cost share in € in year t

$D_{t}$ Distribution factor for the reduction of inefficiencies in year t

${CS}_{c,t}$ Controllable cost share in € in year t

$B_{0}$ Efficiency bonus in € in base year

T Time period of five years

${CPI}_{t}$ Consumer price index in year t

${CPI}_{0}$ Consumer price index in base year

${PF}_{t}$ General sectoral productivity factor in year t

With the model MERLIN, the temporarily non-controllable costs, the controllable costs and the capital costs deduction are determined according to the regulation explained in section 2.2.1. It starts with the determination of the capital cost deduction, and hence with capital costs (equations [Eq. (4)] and [Eq. (5)] in section 2.2.1). The linear depreciation is performed including the time dependencies, meaning that the base year for the third regulation period (2018–2022) is 2015, hence only assets built in and before 2015 are taken into account. This time dependency is considered for all following regulation periods until 2050.

Before the return on equity can be calculated, the deduction capital and the circulating assets need to be calculated for the years of the regulation period. According to experts, this is achieved by aligning their development with the asset development, so that their value in the base year is multiplied with the change of residual values. Equation (7) shows the calculation of the change of residual values and afterwards, the multiplication with the base year values of the deduction capital (equation (8)) and the circulating assets (equation (9)).

[Eq. 7] $${CoR}_{t}=\frac{\sum_{n=1}^{N}{mRV}_{new,n,t}+\sum_{n=1}^{N}{mRV}_{old,n,t}}{\sum_{n=1}^{N}{mRV}_{new,n,0}+\sum_{n=1}^{N}{mRV}_{old,n,0}}$$

[Eq. 8] $${DC}_{t}={DC}_{0}\cdot {CoA}_{t\;}$$

[Eq. 9] $${CA}_{t}={CA}_{0}\cdot {CoA}_{t\;}$$

With:[

${CoR}_{t}$ Change of residual values in year t

${mRV}_{new,n,t}$ Mean residual value of one new asset n in € in year t

${mRV}_{old,n,t}$ Mean residual value of one old asset n in € in year t

n Asset element

N Number of all assets

${DC}_{t}$ Deduction capital in € in year t

${CA}_{t}$ Circulating assets in € in year t

With the deduction capital and the circulating assets in the years of the regulation period the return on equity can be determined, including a check if the maximum share of 40 % is exceeded. In case of overshooting equity, the overshooting share is multiplied with a smaller interest rate, determined by the regulatory authority, than the equity interest rate [13]. Based on the return on equity, the trade tax is calculated and the return on debt in the years of the regulation is adjusted based on the changes of operating assets, which are the sum of residual values from old and new assets as well as the circulating asset. The sum of the depreciation, the return on equity, the trade tax and the return on debt results in the capital costs including the different time dependencies.

To calculate the capital cost deduction the capital costs of the different years in the regulation period are subtracted from the capital cost in the base year ([Eq. (5)] in section 2.2.1). However, the capital costs for 2020, which is the base year for the fourth regulation period, are calculated based on the assets in 2015, the former base year. Hence, in parallel the continuous capital costs need to be determined, including the investments made after the base years. Therefore, the capital cost deduction is the continuous capital costs in the base year minus the capital cost in the regulation period.

Thereafter, the total network costs are adjusted to the asset change, to include the impact of the investment decision. Therefore, the original capital costs (assumed as constant) are removed from the total network costs and the newly calculated continuous capital costs are added. Based on the new total network costs, the capital cost deduction is calculated, and with the model-exogenous permanent non-controllable costs and efficiency factor, the temporary non-controllable cost and the controllable costs are determined according to equations [Eq. 2], [Eq. 3] and (2) in Section 2.2.1. In the end, the different parameters are summed up according to equation (6), resulting in the revenue cap before the start of a regulation period.

To include the changes made after the base year of each regulation period, the capital cost mark-up, the changes in volatile costs and the balance of the regulation account is added to the part of the revenue cap already explained. The changes in volatile costs and the balance of the regulation account depend on the deviations between planned and actual values, which deviate due to external influences such as lower demand than expected. These influences are difficult to simulate and therefore not included in this model. Consequently, only the capital cost mark-up is added to the revenue cap set before the beginning of the regulation period, which is calculated according to the explanations in Appendix B.

#### 3.1.2 Determination of network charges (modul 2)

Network operators have a certain degree of freedom to determine the network charges [13]. These charges are used to distribute the revenue cap approved among the network users, to cover the network costs [36]. point out that the allocation of regulated network cost focuses on cost recovery and not on efficient use of infrastructure. Furthermore, they emphasize the need to increase transparency in cost allocation and data availability. In Germany network operators usually do the distribution to various network levels by means of cost unit accounting and afterwards allocate the cost to the various consumer groups based on their demand. This procedure is highly depending on the network structure and the cost structure of each individual network operator, hence difficult to generalize, underlining the point made by Ref. [36]. Therefore, in this paper only the annual average network charges of the network analyzed (${NC}_{t}$) are calculated by distributing the revenue cap including the capital cost mark-up (${RC}_{t}$) to the model-exogenous demand development ($d_{t}$) (see also section 3.2.1). Equation (10) illustrates the simplified calculation of the network charges with t being the years between 2018 and 2050.

[Eq. 10] $${NC}_{t}=\frac{{RC}_{t}}{d_{t}}$$

#### 3.1.3 Implementation of net present value (module 3)

To provide an insight on how the different regulation options affect the network operator, the net present value (NPV) approach is used. This approach is a common method to evaluate investments in asset management, especially of bigger investment projects, such as energy network investments [37,38]. The NPV is the difference of all discounted revenues ($R_{t}$) and expenses ($E_{t}$) in total, plus the liquidity revenue at the end of the lifetime ($L_{n}$) and minus the investment at the beginning ($I_{0}$) (equation (11)) [38].

[Eq. 11] $${NPV}_{0}=\sum_{t=0}^{n}\frac{R_{t}-E_{t}}{{\left(1+i\right)}^{t}}+\frac{L_{n}}{{\left(1+i\right)}^{n}}-I_{0}$$

With:

i Interest rate

n Lifetime

t Time index 0 until n

The evaluation of the NPV is done for the year 2023, considering annual investments. Hence, there is not one investment at the beginning of the evaluation, but every year. The lifetime included in equation (11) is the lifetime of the network, depending on the chosen scenario. For this paper the scenarios investigated still include the target of climate neutrality until 2050, instead of 2045.^3^

In practice, the result of network charges paid based on the demand needed results in the revenue, which is limited by the revenue cap, as shown in equation (12). However, in this paper a variation between demand planned and actual demand is not considered, so that the revenue cap with capital cost mark-up is used as the revenue.

[Eq. 12] $$R_{t}=\min\left\{d_{t}\cdot {NC}_{t};\;{RC}_{t}\right\}$$

The expenditures are the total network costs included in the revenue cap calculation excluding the capital costs, such as depreciation, return on equity, trade tax and return on debt. The capital costs are already included in the interest rate; hence this prevents their double counting. The equity interest rate for new assets, set by the regulatory authority, is used as interest rate, which includes a market-based risk assessment.

#### 3.1.4 Model validation and representativeness

To evaluate how well the model MERLIN can reproduce real values, the model is applied for the municipal utility Karlsruhe (SWK) and municipal utility Esslingen (SWE). SWK is a vertically integrated energy supplier, which supplies costumers with electricity, natural gas, heat and water [39]. As an unbundled network operator, the municipal utility Karlsruhe network services GmbH (SWKN) are responsible for the networks in Karlsruhe and Rheinstetten. The main network components are leased from the SWK and the network ownership company (Netzeigentumsgesellschaft) Rheinstetten GmbH & Co. KG (NEG). In total the network is 810 km long (status 2021) with 28,612 network connection points [40].

In contrast, SWE is responsible for sales of electricity, natural gas, water and heat, but also for the distribution of natural gas, hence the network operation [41]. SWE owns its network components, which sum up to a 447 km long network (status 2021) with 18,016 network connections. Consequently, it is a rather small network operator compared to SWKN.

In the following the model results of the revenue cap before the third regulation period are compared to the real data for SWKN and SWE (Table 2). These are published by the state regulatory authority of Baden-Württemberg [42]. On the left side the results for SWKN are shown and on the right side the ones for SWE. It can be seen that for SWKN the deviations between calculated and real values are between 0.20 % and 0.14 %, whereas the deviations for SWE are between 0.10 % and 0.08 %.

**Table 2:** Comparison of the calculated and real revenue caps before the beginning of the regulation period for SWKN and SWE (own calculations and data from SWKN/SWE).

Year	Municipal utility Karlsruhe network services			Municipal utility Esslingen		
	MERLIN in k €	SWKN in k €	Difference in %	MERLIN in k €	SWE in k €	Difference in %
2018	24,031	23,984	0.20	12,395	12,404	−0.08
2019	23,522	23,479	0.18	12,148	12,160	−0.10
2020	22,955	22,916	0.17	11,878	11,885	−0.06
2021	22,206	22,174	0.14	11,517	11,526	−0.08
2022	21,473	21,444	0.14	11,163	11,173	−0.08

Additionally, the revenue cap during the third regulation period for SWE and SWKN as well as the temporarily non-controllable costs and the controllable costs for SWKN are publicly available, so they can be evaluated as well. The results are included in Appendix C Table A.7 and Table A.8.

Overall, the validation of the model MERLIN shows that the deviation to reality is less than 1 % in all tests, and hence the model represents the reality quite accurate. Furthermore, by applying the model to SWKN and SWE shows that it is individually useable for different network operators. With 28,612 exit points in Karlsruhe and 18,016 exit points in Esslingen both network operators are classified in the category “> 10,000 & ≤ 100,000 market locations” by the German regulatory authority in its annual monitoring report [27]. 34 % of the German gas distribution network operators included in Ref. [27] are in the same category, while the majority (57 %) are in the category “> 1000 & ≤ 10,000”. Consequently, the gas network operators investigated in this paper have more exit points than most of the gas network operators considered in Ref. [27]. Karlsruhe is, with 810 km, in the network length category "> 500 km & ≤ 1000 km" of [27] and Esslingen, with 447 km, is in the one level smaller category of "> 250 km & ≤ 500 km". The largest proportion of the distribution network operators considered (49 %) are assigned to the "≤ 250 km" category and therefore have significantly shorter networks than the operators analyzed in this paper [27]. Lastly, the incentive-based regulation with a revenue cap is used nationwide in Germany and is the most common regulatory setting for gas distribution network operators in the EU. Therefore, with small adjustments the model developed can be applied in other EU countries.

### 3.2 Data input and assumptions

Due to the fact that most of the data from SWE, SWK, SWKN and NEG underly a non-disclosure agreement, the real data is anonymized to provide a detailed insight into the findings drawn from the investigation with the model MERLIN. Therefore, two network operators are created based on the sum and on the average of the data from SWE, SWK, SWKN and NEG. The results show that the development of values is similar to real data, so that an anonymization is realized, without data distortion. Due to the similarities between the sum-based network operator and the average-based one, in the following the focus is solely on the average-based network operator. All investigations are carried out for both, showing similar findings. More details on the fictitious network operators can be found in Appendix D and in Ref. [32]. The resulting asset portfolio for the average-based network operator is included in Appendix E.

In the following, the gas demand scenarios investigated for the average-based network operator are introduced, the connection between demand development and network length is explained, and the different input data and assumptions for the investment options are described. All costs are values in €_2020_.

#### 3.2.1 Demand scenarios

The demand scenarios in this paper are based on the scenarios in the project TrafoKommunE, which assesses the transformation process for the municipal energy transition funded by the Federal Ministry for Economic Affairs and Climate Actions (10.13039/100021130BMWK) in Germany [43]. Further information about the project is available in Refs. [43,44] and in Ref. [45]. In this project, the demand scenarios are based on the nation-wide long-term scenarios funded by 10.13039/100021130BMWK [46] and scaled-down to the regional level.

**Fig. 3:**
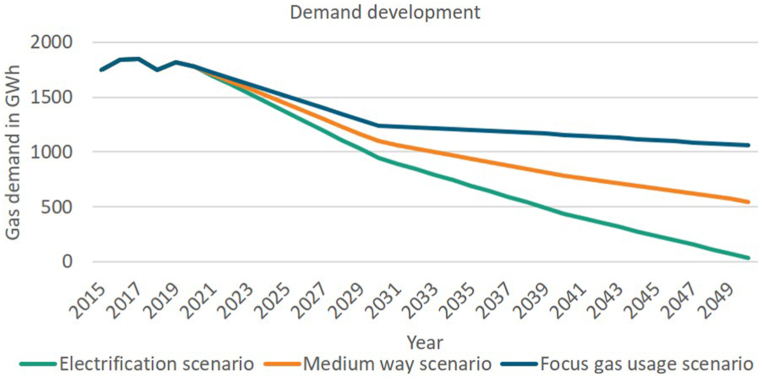
Demand development in project TrafoKommunE [43].

Fig. 3 provides an overview of the demand development in TrafoKommunE. The focus gas usage scenario (blue) are based on the TN-PtG/PtL and assumes more favorable conditions for further demand for gas, while the electrification scenario(TN-Electricity) relies on strong electrification, e.g. through heat pumps [46]. Within TrafoKommunE a medium way scenario (orange) between these two extreme scenarios is determined. In contrast to the TN-Electricity scenario of the long-term scenarios, the regional electrification scenario of TrafoKommunE has a remaining gas demand in 2050 of almost 2 % of today's gas demand, because of the assumption that a few houses cannot be supplied by heat pumps, heating network or other alternatives to gas-based technologies.

#### 3.2.2 Derivation of network length based on demand development

When dealing with a declining gas demand, as the demand development in section 3.2.1 shows, the effects on an existing network structure have to be observed. These decreases can occur by two mechanisms. Frist, a network user switches fuel. In that case, the connection to the gas network is no longer in use. Second, a network user lowers consumption. In that case, the overall demand also decreases, but the connections stay in use. This distinction is important because it describes if a branch of the network becomes obsolete. If all network users in topological proximity depart from the network, that branch may become obsolete, as long as it is not needed to supply remaining network users further downstream [22]. show, that the remaining length of the network for a random distribution of fuel switching network users in real gas distribution networks, can be approximated by a power law function [Eq. (13)].

[Eq. 13] $$\begin{array}{c} L_{t}^{Network}=L_{t=0}^{Network}\;\cdot {\left(\frac{N_{t}^{Network\;user}}{N_{t=0}^{Network\;user}}\right)}^{k}\\ \overset{Subst.}{\to }\;L_{t}^{Network}=L_{t=0}^{Network}\;\cdot {\left(\frac{d_{t}^{Network\;user}}{d_{t=0}^{Network\;user}}\right)}^{k} \end{array}$$

With:

$L_{t}^{Network}$ Network length in year t

$d_{t}^{Network\;user}$ Demand of network users at time t

$N_{t}^{Network\;user}$ Number of network users at time t

k Describes deviation from linear relationship between demand and network length

The dependency of the remaining network length being a function of the decrease in network user numbers, stems from the randomness of network user departure assumed and the topology of low-pressure gas networks in Germany. The strength of this dependency is given by the exponent *k*. The limit of almost complete departure of all but the furthest downstream customers, either by unfortunate topology or external factors, is expressed by *k* approaching 0. In this case almost no obsolete network length can occur, since there are always remaining network users downstream. But it also means, that in such a case with a proportionality of low values of *k*, a network operator has to expect a delayed increase of obsolete assets at first, which in turn may accelerate drastically as more network users depart and the overall length has to approach zero without remaining network users. Higher *k* values (∼1) calculate the case of a more direct impact, such that each departing network user generates nearly the same obsolete length of network as the next one. This correlation may be incentivized by network operators to strategically manage asset lifetimes. For example, network users on an end-of-life approaching branch of a network could be contacted and switched over to a different source of heating as a cluster, if the branch already faces critical levels of demand decrease. Earlier work by Ref. [46] show a scaling of the optimal length, which here could be viewed as source-sink paths, with the number of nodes to the power of 1/3 in weak disordered network types (e. g. Erdös-Rényi and Watts-Strogatz [46]). The investigations from Ref. [22] of values for randomly departing network users ranges in average between 0.4 and 0.5. This might reflect the case of individual decisions of network users to depart from the gas network. To expand our results beyond the assumption of random departures, *k* values of 0.3 and 0.9 are used. With this, the more difficult to manage case for network operators for lower values and the representation of an incentivized progression with higher values, are considered.

#### 3.2.3 Assumptions for the different investment options

Today, the uncertain situation for network operators due to the challenges of the energy transition mentioned before results in gas network operators focusing only on the most needed investments to keep the network running. Therefore, in this paper an investment stop, as an extreme case of not investing anymore, is set as base scenario and compared to different decommission strategies with different regulation options.

##### 3.2.3.1 Investment stop

In the investment stop option, no new investments are made but the network operation is continued. Consequently, the permanent non-controllable costs, including upstream network costs, supplementary wage benefits and costs for vocational training as well as further training stay constant. The input of total network costs stays constant as model-exogenous data and are adjusted model endogenously according to change of capital costs as mentioned in section 3.1.1. This is carried out similarly for circulating assets, deduction capital and return on debt, while it is assumed that prepayments and construction cost subsidies are phased out after 2022 as no new users are connected to the network. All assumptions can be found in Appendix E.

##### 3.2.3.2 Decommissioning

To include the decommission costs into the regulatory framework, three different ways of decommissioning gas pipelines are distinguished. By sealing a pipe, it stays in the ground as a cavity and it is merely inerted [16], meaning the introduction of inert gases, such as nitrogen, to prevent corrosion and explosions [47]. If a pipeline is dammed and sealed, it also stays in the ground but after inerting, it is filled with bentonite, so that no cavity remains. In contrast, when dismantling a pipeline, it is completely removed from the ground and the original condition of the land is restored [16]. Depending on the diameter and material of a pipe the different decommission measures can be applied. Nonetheless, landowners can stipulate in the corresponding right-of-way contracts (concession agreements) the complete dismantling of pipelines, which are not anymore needed for gas distribution [16].

As mentioned in section 2.1, only [16] provides cost assumptions for decommission measures, which are shown together with their shares needed for the German gas distribution network in Table 3.

**Table 3:** Assumptions of shares and costs of the different decommission measures for the German gas distribution network [11,16].

Decommission measures	Share of German gas distribution network in %	Costs in €/km
Dismantling	5	280,000
Damming and sealing	30	70,000
Sealing	65	20,000

Due to the uncertainty of the shares of dismantling, damming and sealing and only sealing, the shares of [16] are used further and investigated within a sensitivity analysis. As landowners can stipulate in their concession agreement that unused pipelines need to be removed from the ground, this leads to an extreme case for the network operator, as it leads to the highest decommissioning costs and therefore is further investigated in this paper. Overall, in this paper we distinguish two decommission strategies, the fully dismantling of the network and the partly decommissioning based on the shares of decommissioning measures according to Ref. [16].

**Fig. 4:**
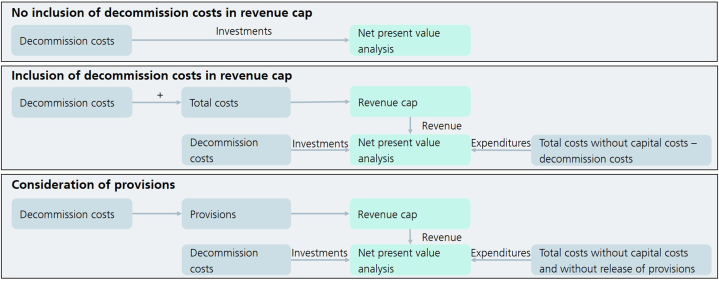
Illustration of implementation of the different regulation options in the model MERLIN (own illustration).

For the decommission strategies, different regulation options are implemented, as explained in section 2.3. In case that the network length reduction of one of the scenarios demands the decommissioning of pipelines not yet depreciated, their lifetime is reduced, so that all pipelines are fully depreciated by the time of decommission. Fig. 4 provides an overview of the implementation of the different regulation options in the model MERLIN.

To investigate the option of not including the decommission costs (first option in Fig. 4), these are solely evaluated as investments in the NPV analysis. For the regulation option of fully including the decommission costs (second option in Fig. 4), these are added to the total network costs adjusted to the capital cost development, so that the revenue caps resulting include these costs. Consequently, before calculating the NPV these costs are removed from the expenses considered and are included as investments to evaluate.

Lastly, for the consideration of provisions (third option in Fig. 4) the time dependencies of the regulation periods are important. In the above-mentioned options, the network operator decides in 2023 to decommission and can already start the decommission in 2024 as soon as the pipelines are not needed anymore. If this decommissioning should be financed with provisions, these need to be earned before and consequently included in the regulation before. Provisions are part of the deduction capital included in the capital cost determination. Therefore, only in the base year provisions are considered, leading to smaller capital cost deductions, and hence to higher revenue caps and higher network charges. After the provision is earned and the decommissioning starts the release of provision is included in the total network costs, leading to lower costs. Therefore, the network operator decides to decommission in 2023, so in 2025, 2030, 2035 and 2040 provisions are included of the same amount needed for decommission in the next regulation period. This means that the provision in 2025 corresponds to the decommission costs needed for the decommissioning between 2033 and 2037, which is the next regulation period after the provisions are earned. During that regulation period, the provisions for the next decommission phase in the next regulation period are earned, and so on. This leads to a certain time delay, so that in the first regulation period after the decommission decision higher network costs are included than in the regulation option of not including or including decommission costs in the revenue cap.

In all demand scenarios considered, at least a slight demand is still left in the network and needs to be supplied. Consequently, the permanent non-controllable costs stay constant. In contrast, the operational costs, such as material and personnel expenditure, are assumed to be dependent on the network length and consequently decrease accordingly. This decrease is also taken into account for the total network costs. The assumption taken is well suited for the material expenditure, however a decrease of the personnel expenditure according to network length is a very optimistic view for the network operator and hence, is further varied in a sensitivity analysis.

Lastly, for the circulating assets and the return on debt as well as the prepayments and the construction cost subsidies the same assumptions are taken as for the investment stop option.

## 4 Results

In the following, the results providing insight into the impact of different investment strategies on the network operator and user, are outlined and explained. It starts with the investment stop option, as the base case. Afterwards the different network lengths, derived from the power function explained in section 3.2.2, are illustrated and then the impact on the network operator and user is shown by depicting its effect on the NPV and the network charges. Lastly, the resulting capital cost deductions shows the limitation of the current regulatory framework in Germany.

### 4.1 Investment stop

In this option, the average-based network operator stops taking any investments as an extreme case of the current situation. Even though only the already existing network components are included in this analysis, in 2050 it will result in remaining high residual values, which can be “stranded assets” if a strong demand reduction occurs. For the average-based network operator, the residual values result to 20 million € in 2045 and 13 million € in 2050. More detailed insights are provided in Appendix F and [32].

**Fig. 5:**
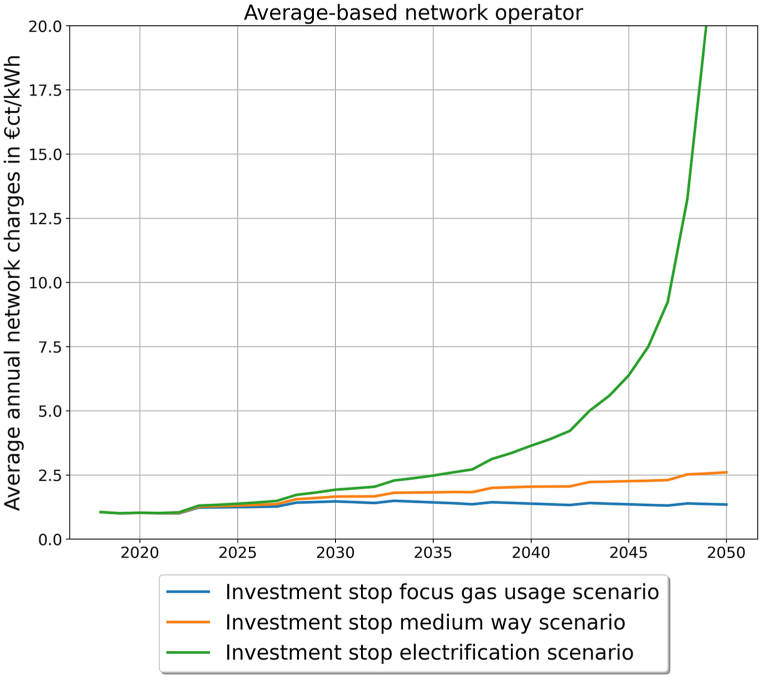
Network charges for the different demand scenarios for the average-based network operator (own illustration).

With the network charges the impact of the investment option and the demand development on the network user can be analyzed. Fig. 5 shows the development of the network charges in the different demand scenarios for the average-based network operator. One may observe that network charges stay nearly the same for the gas scenario, while for the medium way scenario the network charges are more than double the current network charges in 2050 (from 1.02 €ct/kWh in 2020 to 2.60 €ct/kWh in 2050). In the electrification scenario the network charges grow exponentially up to around 45 €ct/kWh in 2050. In comparison, the price of natural gas for household costumers was 6.68 €ct/kWh in 2021 [27]. Due to the Ukraine war, it rose to 22 €ct/kWh in September 2022, but is currently falling again [48].

This strong increase in network charges in the electrification and medium way scenario illustrates the need to reduce the size of the gas distribution network by decommissioning. In this paper the gas demand developments are the base to derive the impact of different decommission strategies and their regulation options, to see if there is a different impact if the gas demand development is different. A direct comparison between the gas demand scenarios is not part of this analysis.

### 4.2 Decommissioning strategies

To investigate the various decommissioning strategies, the network lengths are derived as a first step. These are calculated according to section 3.2.2 based on the demand development shown in section 3.2.1. Afterwards, the NPV of the decommission strategies and the different regulatory options show the impact on the network operators. With the network charges, these effects are analyzed for the network user and in the end the limits of the current regulation are shown by investigating the capital cost deduction.

#### 4.2.1 Network length

**Fig. 6:**
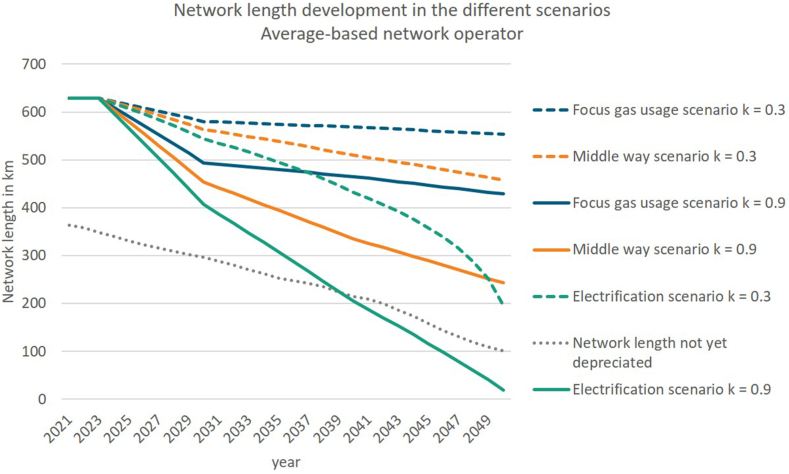
Resulting network length based on power function and demand development (own illustration).

Out of the three demand scenarios, six network length scenarios result, based on the approach described in section 3.2.2. Fig. 6 depicts the network development for the case, that in 2023 the network operator decides to start decommissioning as soon as a pipeline is not needed anymore. This type of decommission strategy is further included in the regulation options of not including and including the decommissioning costs in the revenue cap. Depending on the different k-values and demand development a broad variation of network lengths resulted. While the network length only slightly decreases in the focus gas usage scenario with k = 0.3, meaning a low linearity between demand development and network length, the length in the electrification scenario k = 0.9, with a high linearity between demand development and network length, decreases significantly. The grey dotted line (Fig. 6) illustrates the network length not yet fully depreciated, and hence only in the electrification scenario k = 0.9 network components are not anymore needed, even though they are not yet refinanced by network charges. Overall, this approach only investigates the network length reduction by quantity, not by location. Consequently, in reality even in the focus gas usage scenario k = 0.3 network components might not be needed anymore, even though they are not depreciated, due to the location, in which network users change to other non-gas-based technologies.

**Fig. 7:**
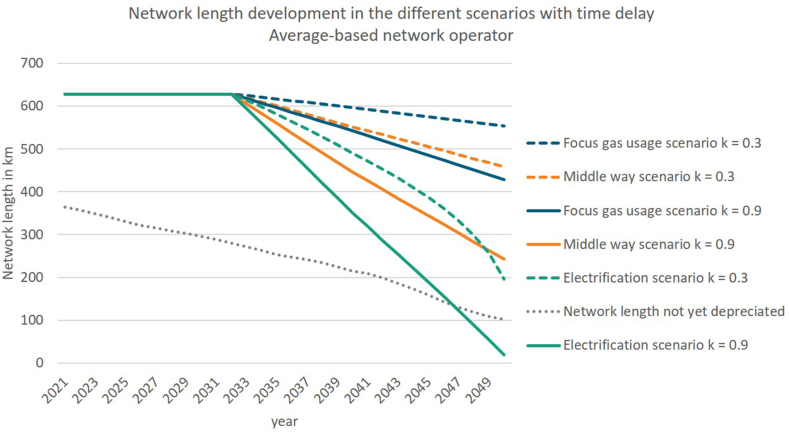
Network length development with delay due to provisions (own illustration).

In contrast to Figs. 6 and 7 includes the time delay in network length reduction, due to the consideration of provision, explained in section 3.2.3.2. Therefore, in 2023 the decision to include provisions for a later decommission is taken into account in the revenue cap and after 2033 the actual network length reduction starts. This reduction is steeper than the one in Fig. 6, because in 2050 the same length of the gas network remains in the different scenarios as before.

#### 4.2.2 Impact on network operator

The impact on the network operator of the different decommission strategies, and the different regulation options is shown by NPVs. Table 4 compares the effects within the electrification scenario k = 0.3. For the partial decommissioning strategy, the inclusion of the decommissioning costs leads to a slight positive NPV, whereas the consideration of provision is slightly resulting in lower losses than in the case of no decommission costs considered in the revenue cap. Similar results are seen for the full dismantling, with the difference that no NPV achieves a positive value, due to the high decommission costs and the network operator considered in this study has an efficiency factor of 86 %. Consequently, it is harder to achieve a positive NPV compared to an efficient network operator with an efficiency factor of 100 %. Further, Table 4 includes the NPV of the investment stop strategy, which results in higher losses than the partial decommissioning but far lower losses than the full dismantling. Hence, not doing anything would be preferable over full dismantling of the network from a network operator perspective. Lastly, the bottom row includes the remaining value of the network in 2050.

**Table 4:** NPV for the different investment strategies in the electrification scenario k = 0.3 (own illustration).

Impact on network operator (NPV in thousand € in 2023)	No inclusion of decommission costs in revenue cap	Inclusion of decommission costs in revenue cap	Consideration of provisions
Investment stop	−8652		
Electrification scenario k = 0.3 full dismantling	−48,933	−13,119	−20,260
Electrification scenario k = 0.3 partly decommissioning	−5424	715	−3716
Residual value of the network without reduction of depreciation period	3495		

For the electrification scenario k = 0.9, meaning a nearly linear development of network length and demand, the results are similar as for the electrification scenario k = 0.3, with more extreme values (Table 5). One difference to the beforementioned scenario results is that including the decommissioning cost leads to losses of only minus 4.6 million €, which is less than for the investment stop. Hence, even full dismantling with including the decommission costs is better than not doing anything. Furthermore, the shortened depreciation period in this scenario results in slightly lower residual values than for the other scenarios, however the time delay due to the consideration of provision has only a slight influence on the residual value in 2050.

**Table 5:** NPV for the different investment strategies in the electrification scenario k = 0.9 (own illustration).

Impact on network operator (NPV in thousand € in 2023)	No inclusion of decommission costs in revenue cap	Inclusion of decommission costs in revenue cap	Consideration of provisions
Investment stop	−8652		
Electrification scenario k = 0.9 full dismantling	−81,466	−4629	−27,389
Electrification scenario k = 0.9 partly decommissioning	−749	12,423	−16
Residual value of the network without reduction of depreciation period	3495		
Residual value of the network with reduction of depreciation period	2217		
Residual value of the network with reduction of depreciation period and consideration of provision	2218		

For the medium way and gas scenarios the results are comparable to the electrification scenarios, with the difference that for the k = 0.3 scenarios only negative NPVs are achieved and the differences between the losses and profits are lower than for the electrification scenarios. Therefore, the tables for these scenarios are attached in Appendix G.

#### 4.2.3 Impact on network users

**Fig. 8:**
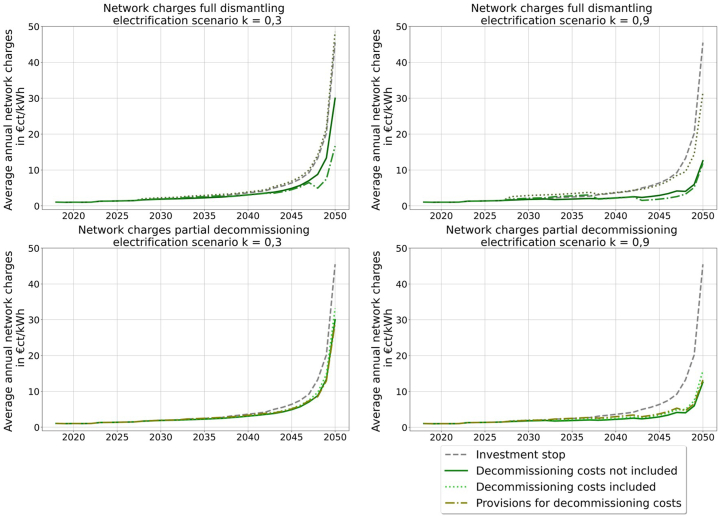
Network charges full dismantling and partial decommissioning of the electrification scenario (own illustration).

With the resulting network charges the influence of the different decommission strategies and regulation options on the network user are illustrated. Fig. 8 shows the resulting network charges for the electrification scenario. In the upper left corner, the network charges for full dismantling in the electrification scenario k = 0.3 are shown. The option of including the decommissioning costs results in the highest network charges, which are even higher than for the investment stop. In contrast, the consideration of provisions leads to the lowest network charges and hence, is favorable for the network users. A similar conclusion can be drawn from the network charges for full dismantling in the electrification scenario k = 0.9 (Fig. 8 upper right), even though there is only a small difference between considering provisions and not including any decommissioning costs. The lower two graphs in Fig. 8 show the same scenarios with partial decommissioning. Overall, there are only small differences between the different regulation options, and the effect of considering provisions cannot be observed, meaning higher network charges between 2027 and 2037, as well as lower ones afterwards. This is mainly due to the longer high network costs, because of the time delay of taking provisions into account before decommissioning. This effect is less for full dismantling, because of the far higher decommission costs.

**Fig. 9:**
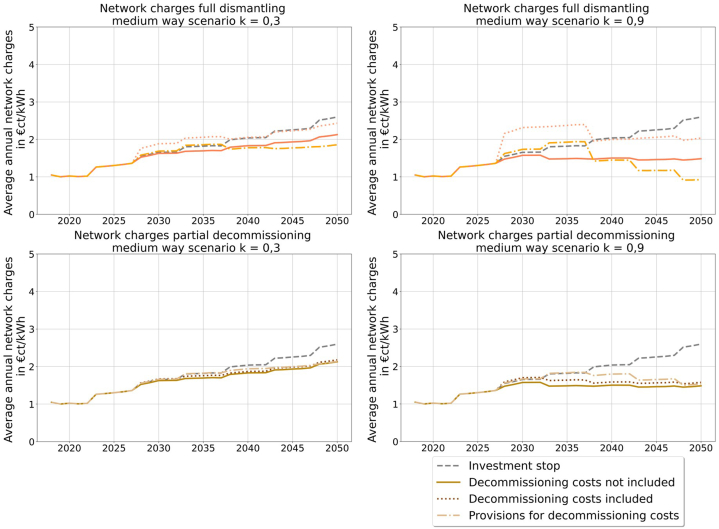
Network charges full dismantling and partial decommissioning of the medium way scenario (own illustration).

In the medium way scenario overall (Fig. 9), the network charges are lower than in the electrification scenario by a factor of 10, so that the changes between the regulation option have a lower overall impact on the price the network user is paying in the end. The variation of the resulting network charges for full dismantling is between 1 and 2 €ct/kWh and for partial decommissioning they vary less than 1 €ct/kWh.

Similar results with even lower variations in network charges result in the gas scenario. The figures can be found in Appendix H.

#### 4.2.4 Resulting capital cost deduction

To look into more detail into the context of the regulation formula, described in section 2.2.1 and 3.1.1, the capital cost deduction is investigated. The consideration of provision leads to a reduction in capital cost deduction, and consequently, to an increase in revenue cap. This can be seen for the partial decommissioning strategies as well as the full dismantling strategies of the gas scenarios and the medium way scenario k = 0.3. In contrast, the electrification scenarios and the medium way scenario k = 0.9 with full dismantling result in negative capital cost deductions, which are not allowed in the current regulatory framework in Germany, because it acts as a bonus on top of the revenue cap, leading to higher revenues allowed, and hence incentivizing higher decommissioning costs. However, setting capital cost deduction to zero leads to higher losses for the network operator, only slightly different network charges, and nearly no cost reduction during the regulation periods. Hence, the decommissioning costs cannot be financed through provision in these scenarios. More details are shown in Appendix I and [32].

### 4.3 Sensitivities

**Fig. 10:**
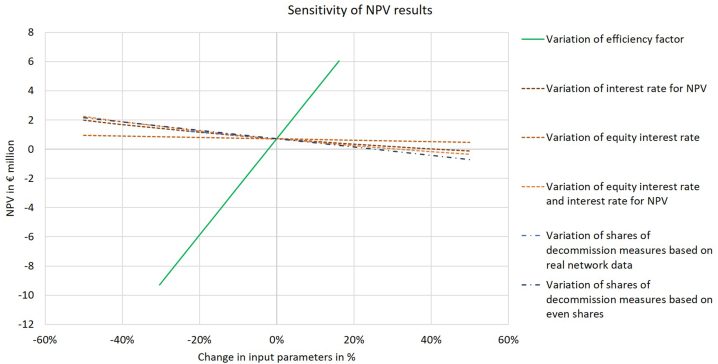
Sensitivity of NPV in 2023 (own illustration).

The high amount of input data needed and their assumptions, explained in section 3.2, include a considerable level of uncertainties, which is further accessed by looking into various sensitivities. One parameter, depending not only on the performance of the network operator analyzed in this paper, but also on similar network operators, is the efficiency factor. The efficiency factor can vary between 60 % and 100 %, so that this range in 10 % steps is part of the sensitivities considered. In the original calculations a constant efficiency factor of 86 % is assumed. The results show a significant influence of the efficiency factor on the NPV (Fig. 10 green line), exemplary for the electrification scenario k = 0.3 including the partial decommissioning costs in the revenue cap. An efficient network operator, with an efficiency factor of 100 %, can reach a profit of 6 Mio. € instead of 0.7 Mio. € (with the original efficiency factor). However, already a reduction of the efficiency factor to 80 % (from 86 %) leads to a negative NPV of 1.6 Mio. €, resulting in a decrease of NPV of more than 100 %. To what extent the decommissioning of gas network pipelines influences the efficiency factor is unclear and depends on the decisions of the other similar network operators in the efficiency comparison.

Another relevant parameter is the equity interest rate for old and new assets. The results so far are based on a 5.07 % interest rate for new assets and 3.51 % for old assets. These interest rates remain constant until 2050. To analyze its sensitivity on the NPV the interest rates are varied between 1 % and 10 % with 1 % steps. Its influence is shown in Fig. 10 light brown dashed line, which depicts a nearly constant line. Nevertheless, a slight increase in the interest rate of one percentage point already leads to a decrease in the NPV of nearly 14 %. Furthermore, the interest rate for new assets is used as the interest rate for the NPV calculation and its variation is shown by the dark brown dashed line in Fig. 10. Its influence is stronger than the one of the interest rates for old and new assets, hence an increase of one percentage point of the interest rate leads to a reduction of NPV of almost 50 %. Lastly, if all interest rates are adjusted, the ones for old and new assets and the one for the NPV calculation, an increase of one percentage point results in a decrease of NPV by 60 % (orange dashed line Fig. 10). Consequently, increasing interest rates lead to lower NPVs and decreasing interest rates to higher NPVs, hence the attractiveness of decommissioning can be influenced by the interest rates.

As mentioned in section 3.2.3.2, the shares of decommission measures for the partial decommissioning depend on the pipeline diameter. However, no clear definition exists to specify the diameter for the different decommission measures. Based on expert opinions from DVGW it is assumed that pipelines with diameters lower than 100 mm can be sealed, while between 100 mm and 500 mm damming and sealing is needed. Dismantling is assumed for diameters higher than 500 mm. The dismantling of previously dammed and or sealed pipes should be combined with other trenchwork to moderate costs. For the network investigated in this paper the exact diameters are unknown, so that different shares of decommissioning measures are investigated according to the assumptions in Appendix J. The light blue and dark blue dash-dot lines in Fig. 10 illustrate their influence on the NPV. It can be seen that the influence of the different shares of decommissioning measures is stronger than the interest rates, but far lower than the efficiency factor. Consequently, the network structure and the decommission measures chosen have a significant influence on the economic feasibility of decommissioning.

The assumption of the personnel costs being according to the network length in section 3.2.3.2 was very optimistic. This assumption leads to a decrease in personnel cost of 70 %, leaving only 30 % included in the calculations. Therefore, the personnel costs are varied from 30 % to 120 % of the original personnel cost in 30 % steps, as well as a consideration of constant personnel costs based on the year 2020. The reason for higher personnel costs could be an increase in salary. Table 6 shows the effect of higher personnel costs on the NPV. Already 30 % more personnel costs in 2050 lead in the electrification scenario k = 0.3 with inclusion of the partial decommissioning costs to a negative NPV. Overall, the higher the personnel cost the lower the NPV, the higher the losses respectively.

**Table 6:** Sensitivity of NPV in 2023 with varying personnel costs (own illustration).

	Proportionate change in personnel costs	NPV in million €
Personnel costs = 120 %	300 %	−10.3
Personnel costs = constant	233 %	−7.3
Personnel costs = 90 %	200 %	−5.8
Personnel costs = 60 %	100 %	−1.2
Personnel costs = 30 % (depending on length)	0 %	0.7

The above explained sensitivities are also carried out for the network charges and revenue cap in 2050, resulting in similar effects as seen for the NPV, however on a far smaller scale. Consequently, the parameters investigated have a stronger influence on the NPV than on the network charges and revenue cap. The corresponding figures are included in Appendix J.

## 5 Discussion

**Fig. 11:**
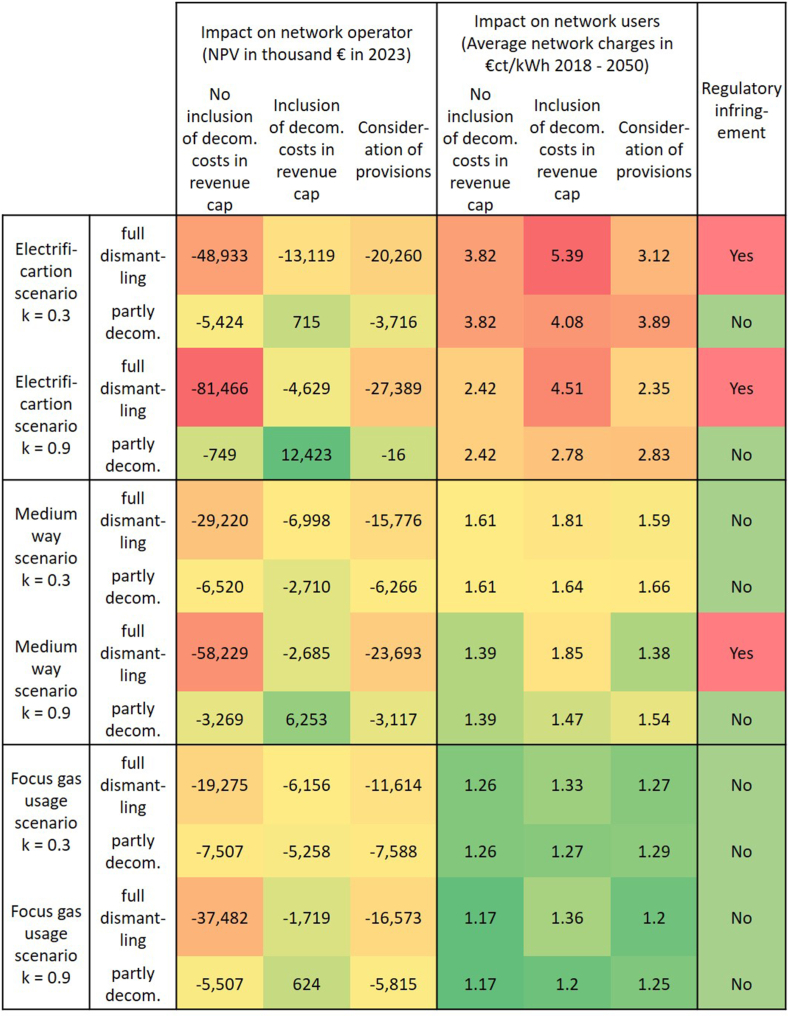
Overview of the impact of different decommissioning strategies and regulation options on network operator and users (own illustration).

To sum up the findings for all scenarios made by the investigation in this paper, Fig. 11 provides an overview of the impact of the different decommissioning strategies and regulation options on the network operator and its users. Scenarios with k = 0.3 have a decrease in gas demand with a non-linear dependence between network length and demand development. In reality, this development could appear, when in a network region many users switch individually and uncoordinated, while on different pipelines still a few downstream network users stay, so that the pipelines cannot be decommissioned. In contrast, for scenarios with k = 0.9 the network length decreases almost linearly to the demand development, which could be the case for an organized phase out of the gas distribution network with external factors. For example, with incentivization of clustered network users by the network operator such as a planned expansion of heating networks. However, a switch from gas distribution network to heating network most likely would lead to length reduction with steps rather than continuous reduction. For the impact on the network operator Fig. 11 compares the resulting NPVs with a color scheme, ranging from high NPVs in green to low NPVs in red. The impact on network users is summarized with the average network charges between 2018 and 2050 and the color scheme ranges from red as the highest network charges to green for the lowest. Lastly, the right column illustrates if negative capital cost deductions appear.

For the network operator in the case of full dismantling the inclusion of the decommissioning costs would result in the most favorable conditions in all scenarios, however for the network user this leads to significantly higher network charges. A compromise could be achieved by considering provisions, leading to higher losses for the network operator, but far lower network charges for the user. Nonetheless, this regulation option leads to negative capital cost deductions in both electrification scenarios and the medium way scenario k = 0.9. In all scenarios with partial decommissioning positive or the lowest negative NPVs appear by including the decommissioning costs in the revenue cap, and only slightly higher or even lower network charges for the network users, so that this option would be the preferred one.

Overall, for the network charges there is a clear tendency towards the higher the remaining gas demand in the scenario, such as for the focus gas usage scenario, the lower the network charges. In contrast, this dependency is not seen for the network operator. For the network operator, i. e. for the NPV, the stronger the gas demand decrease the bigger the difference between losses and profit, consequently the electrification scenario results in the highest profit for the network operator in case of partly decommissioning with including the costs in the revenue cap and a nearly linear alignment between demand and network length, whereas in the same scenario with full dismantling and not including the decommissioning costs in the revenue cap the highest losses appear. Similar findings could be observed for the sum-based network operator.

The approach used in this paper needs a high amount of input data and assumptions, leading to a certain level of uncertainty, and therefore to the need of a sensitivity analysis. Its results mainly illustrate a higher influence of the relevant parameters on the NPV than on the network charges. Especially the efficiency factor remains as one key parameter to change the situation for the network operator drastically. A further research demand could be identified for the influence of decommission strategies on the efficiency factors. Further, the infringement of regulation in the regulation option of considering provisions for full dismantling leads to a demand in regulation adjustment, if the government decides to focus on a rather electrification scenario-based energy system.

The model currently is focusing on the regulation framework tailored to the German regulatory framework. Due to the broad application of incentive-based regulation with revenue cap in the EU, the model can be applied to other countries with small adjustments, such as adjusting the length of the regulation period. However, one important limitation of this work is the lack of location reference of the pipelines being decommissioned. This approach gives a broad overview of different length developments, while including the network structure and certain slowing down effects on the network length reduction with a power function. In reality, even in a scenario with high remaining gas demand, newer not yet depreciated pipelines may become obsolete, leading to higher losses for the network operator. Further, the time delay for including provisions is part of the manufactured decommission strategy, but it is questionable that a network operator would still run pipelines even though there is no remaining demand. Moreover, the network charges provide insights into the effect on the network user, however these charges are just one element of the price paid by end users. Consequently, the influence of the network charges on the end users depends on the overall price development. A strong increase in gas production costs, for example to produce synthetic methane, will lower the effect of strong increasing network charges, while with nearly constant production costs, a significant increase in network charges has a strong effect on the end user. Lastly, in this paper the last end-users are still supplied by the gas network operator. In case of a strong demand reduction the last end-users might need support or incentives to change to an alternative energy supply. Further research is needed into how to design this support or incentive.

## 6 Summary and conclusion

This paper contributes to the scientific community, to political and regulatory decision makers and to the gas network industry by providing insights into the impact of different decommission strategies and regulation options on gas network operators and users, answering the research question: “What effect do different regulations for decommissioning of gas distribution networks have on operators and users?“. This question is analyzed against the background of scenarios with decreasing gas demand at different heights.

With the new model MERLIN, first the German regulatory framework is included in detail, to model gas network operator investment assessment as realistically as possible in cooperation with real gas network operators. This can be used to analyze the economic effects of regulation, which in Germany are determined by a fixed revenue cap for network operators. Based on the revenue cap derived, the network charges are determined and lastly, net present value (NPV) calculations are used to illustrate the effect of the different decommission strategies and regulation options on the network operator and users. This new method allows the integration of current and future regulations in long-term investment analysis, taking into account the time delays from the regulatory settings and long-term demand scenarios. This increases the applicability of the results, which according to Ref. [4] is a gap in the existing literature and has been little explored so far.

### 6.1 Investment stop case illustrates need to adjust depreciation period of existing gas network assets

The first strategy investigated is the investment stop case, which is used as base case, illustrating in an extreme manner the current situation of network operator's uncertainty to invest in the gas distribution network. It shows that with the currently existing network assets, a high number of residual values remain in the gas network considered until 2045 and 2050, which are in danger of becoming stranded assets if gas demand decreases significantly. This leads to the conclusion that shortening of the depreciation period of existing gas network assets is needed. Moreover, a significant reduction in gas demand results in an exponential increase in network charges especially for the last remaining gas consumers. Furthermore, the analyses show that network decommissioning must be planned well and in the long-term, because a situation with few users in a large-scale distribution network cannot be financed.

### 6.2 Partial decommissioning with including the decommission costs is the most economical option

Furthermore, our analysis shows that partial decommissioning, including the decommission costs in the legally fixed revenue cap, is the most economical option for network operators and users. We further see the same regulation option leads in the case of full dismantling in scenarios with high gas demand reduction to the highest profit for the network operator and the highest cost for the network users. Financing the full dismantling by considering provisions leads to negative capital costs deductions, which are not permitted in the current German regulation. Hence, financing high dismantling costs through the consideration of provision is not possible nowadays. Should a full dismantling of the gas distribution networks be enforced politically, then a reform of taking provisions into account in the current regulation is needed. Lastly, the analysis shows the significant influence of the efficiency factor, which is intended to ensure that the gas networks are constructed and used as economically as possible by comparing similar network operators with each other, on the economic feasibility of investment options. Up to now it is unclear how decommissioning gas networks will affect the efficiency comparison and hence, the efficiency factor. Therefore, a separate consideration of decommissioning in the efficiency comparison should be investigated in order to avoid disadvantages due to network decommissioning.

### 6.3 Political decisions on strategic direction are needed

Overall, political decision makers need to decide on their energy transition strategy. If gas demand falls only moderately the regulation option of including the decommission costs suits the best for the decommission regulation, even in most cases if full dismantling is required. However, the path of vast electrification which leads to a drastic drop in gas demand, especially with full dismantling requirements, leads to a dilemma between what is best for network operators and users. Therefore, the German regulatory framework of today will reach its limits in such a case and further research for its adjustment is needed.

### 6.4 Applicability of the method to other countries

In many countries, gas distribution networks are an important infrastructure for supplying the building sector in particular. While the United Kingdom have already adopted bans on gas boilers in new buildings, this is strongly discussed in Germany. Some countries discuss variations for regulating the decommissioning of gas distribution networks and how to deal with “stranded assets”. Even some initial pilot projects for decommissioning are conducted, such as in Switzerland. A shortening of depreciation periods is already practiced in New Zealand and the United Kingdom, similar to the decided shortening in Germany. In most EU countries, it is not yet clear how the decreasing gas demand and the obsolete gas distribution networks will be dealt with in regulatory terms [52]. This paper provides insights that are also relevant for other EU countries with declining gas demand and can support the design of their regulatory framework.

## CRediT authorship contribution statement

**Stella Oberle:** Writing – original draft, Visualization, Validation, Project administration, Methodology, Investigation, Formal analysis, Data curation, Conceptualization. **Till Gnann:** Writing – review & editing, Conceptualization. **Louis Wayas:** Writing – original draft, Methodology, Data curation. **Martin Wietschel:** Writing – review & editing, Supervision, Funding acquisition, Conceptualization.

## Data availability

The asset portfolio of the average-based network operator and the initial input data valid for all investment options investigated have been deposited at Fordatis - Research DataRepository of the Fraunhofer Gesellschaft (https://fordatis.fraunhofer.de/?locale=de) with0-14736-3840-00002.

## Funding

This work is part of the project “Transformation process for the municipal energy transition - TrafoKommunE” funded by the German 10.13039/100021130Federal Ministry for Economic Affairs and Climate Action (10.13039/100021130BMWK, former 10.13039/501100006360Federal Ministry for Economic Affairs and Energy (BMWi)) (project no. (FKZ): 03EN3008A); however, it does not represent the position of the 10.13039/100021130BMWK.

## Declaration of competing interest

The authors declare that they have no known competing financial interests or personal relationships that could have appeared to influence the work reported in this paper.

## Figures and Tables

**Table A.7:** Comparison of the calculated and real revenue caps during the regulation period for SWKN and SWE (own calculations and data from SWKN/ SWE)

Year	Municipal utility Karlsruhe network services			Municipal utility Esslingen		
	MERLIN in k €	SWKN in k €	Difference in %	MERLIN in k €	SWE in k €	Difference in %
**2018**	25,986	25,873	0.44	13,177	13,222	−0.34
**2019**	25,753	25,732	0.08	13,326	13,283	0.33
**2020**	25,805	25,764	0.16	13,348	13,327	0.16

**Table A.8:** Comparison of the calculated and real temporarily non-controllable costs and controllable costs (own calculations and data from SWKN)

Year	Temporarily non-controllable costs			Controllable costs		
	MERLIN in k €	SWKN in k €	Difference in %	MERLIN in k €	SWE in k €	Difference in %
**2018**	18,023	17,981	0.24	2902	2896	0.22
**2019**	17,859	17,820	0.22	2876	2870	0.21
**2020**	17,688	17,652	0.21	2848	2843	0.19
**2021**	17,527	17,496	0.17	2822	2818	0.16
**2022**	17,372	17,344	0.16	2798	2793	0.16

**Table A.9:** Combination of data for anonymization (own illustration)

Input data	Sum-based	Average-based
**Trade tax rate**	Average of SWE, SWK, SWKN and NEG	
**Efficiency factor**	Average of Karlsruhe and Esslingen	
**Asset portfolio, Investments**	Sum of SWE, SWK, SWKN and NEG	Average of assets with same type, purchase year and lifetime
**Construction cost subsidies, circulating assets, deduction capital, other assets, demand, return on debt, prepayments, permanent non-controllable costs, total network costs**	Sum of SWE, SWK, SWKN and NEG	Average of SWE, SWK, SWKN and NEG
**Distribution of inefficiencies, equity interest rate, overshooting equity interest rate, mixed interest rate, general productivity factor, assessment rate, consumer price index**	Same for all network operators	

**Fig. A.12:**
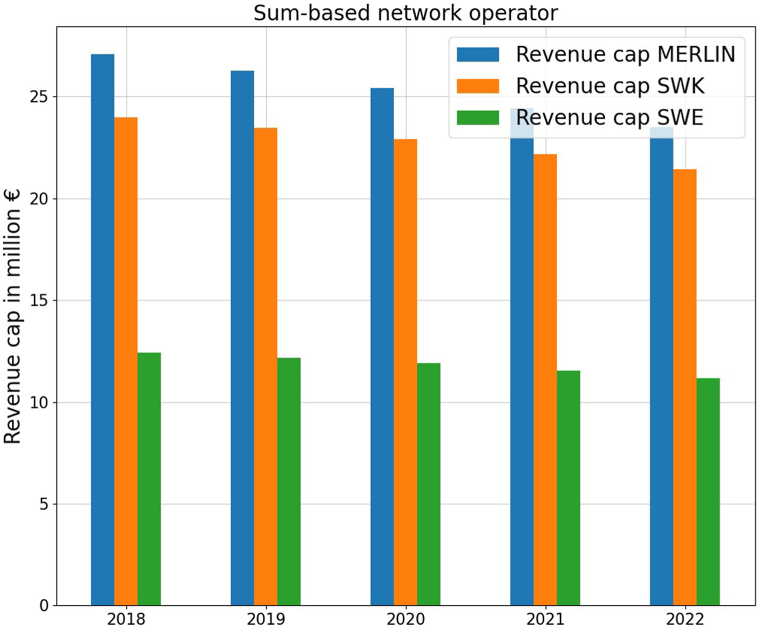
Revenue cap sum-based network operator (own illustration).

**Fig. A.13:**
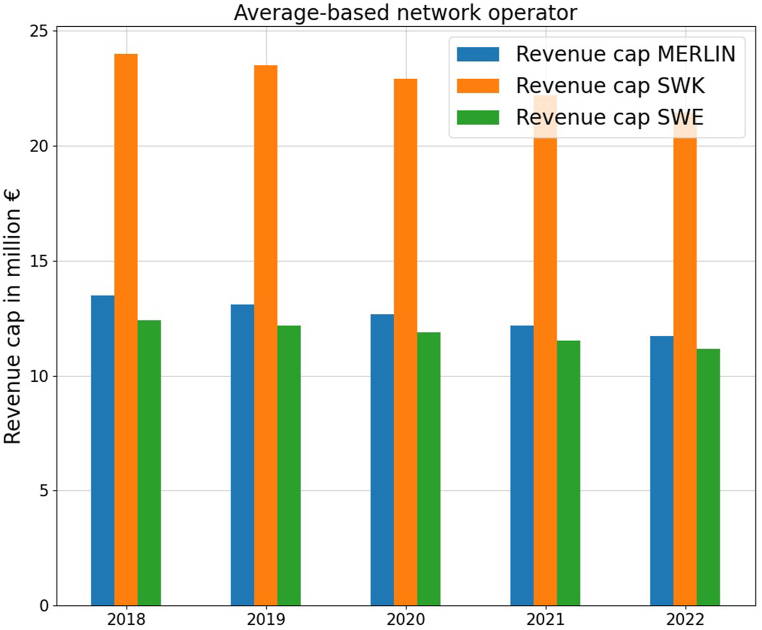
Revenue cap average-based network operator (own illustration).

**Fig. A.14:**
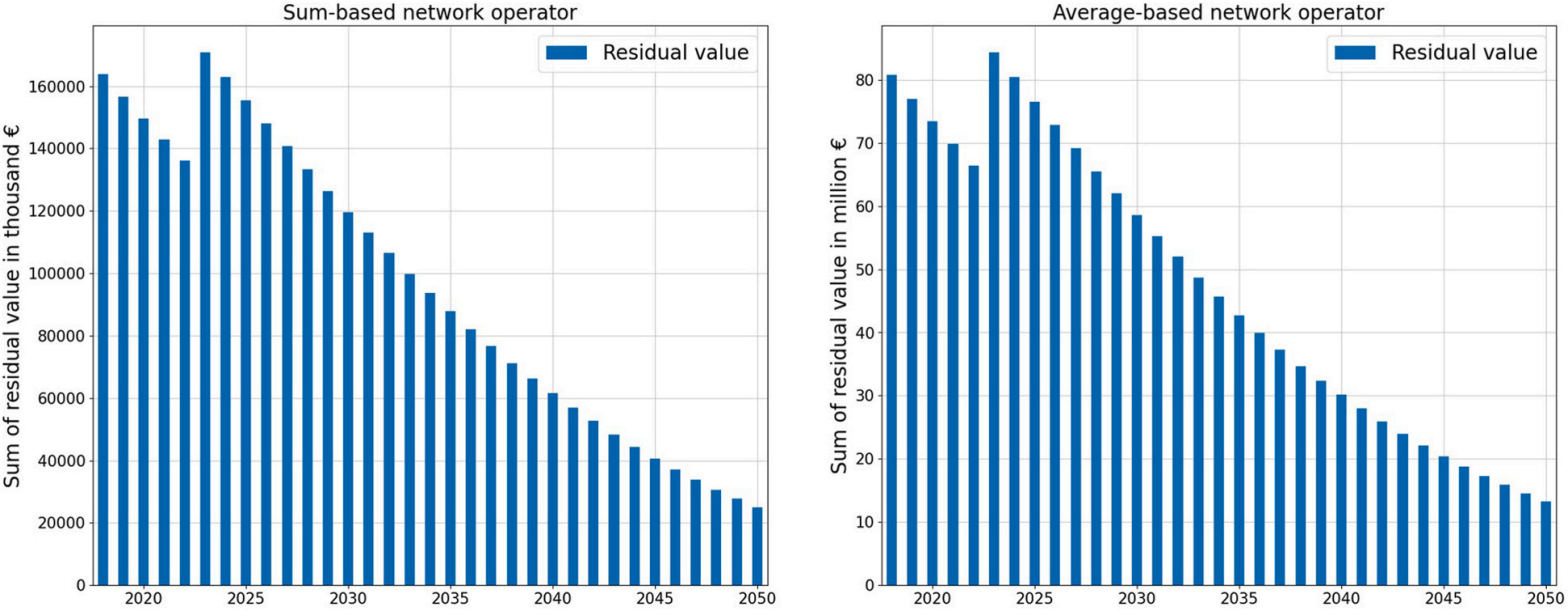
Residual values for sum-based and average-based network operator (own illustration).

**Fig. A.15:**
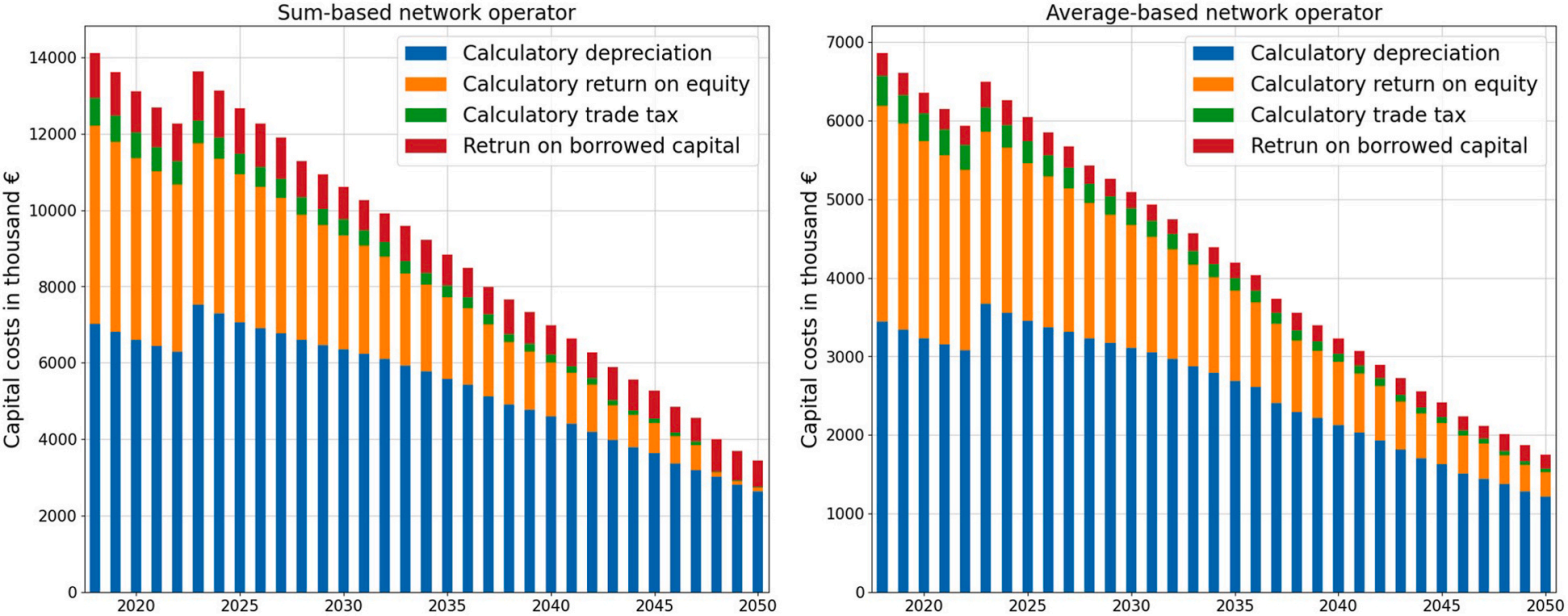
Capital costs of sum-based and average-based network operator (own illustration).

**Fig. A.16:**
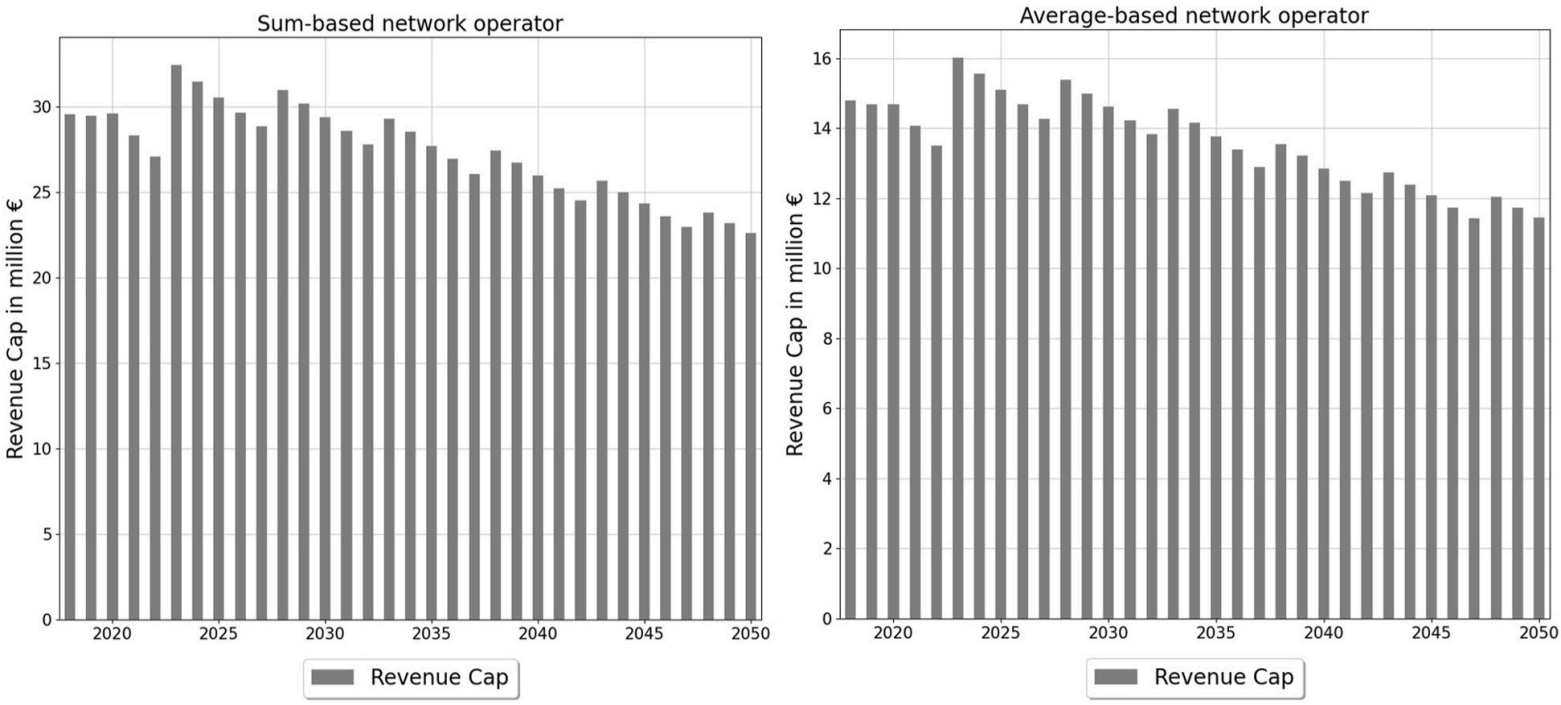
Revenue cap with capital cost mark-up for sum-based and average-based network operator (own illustration).

**Fig. A.17:**
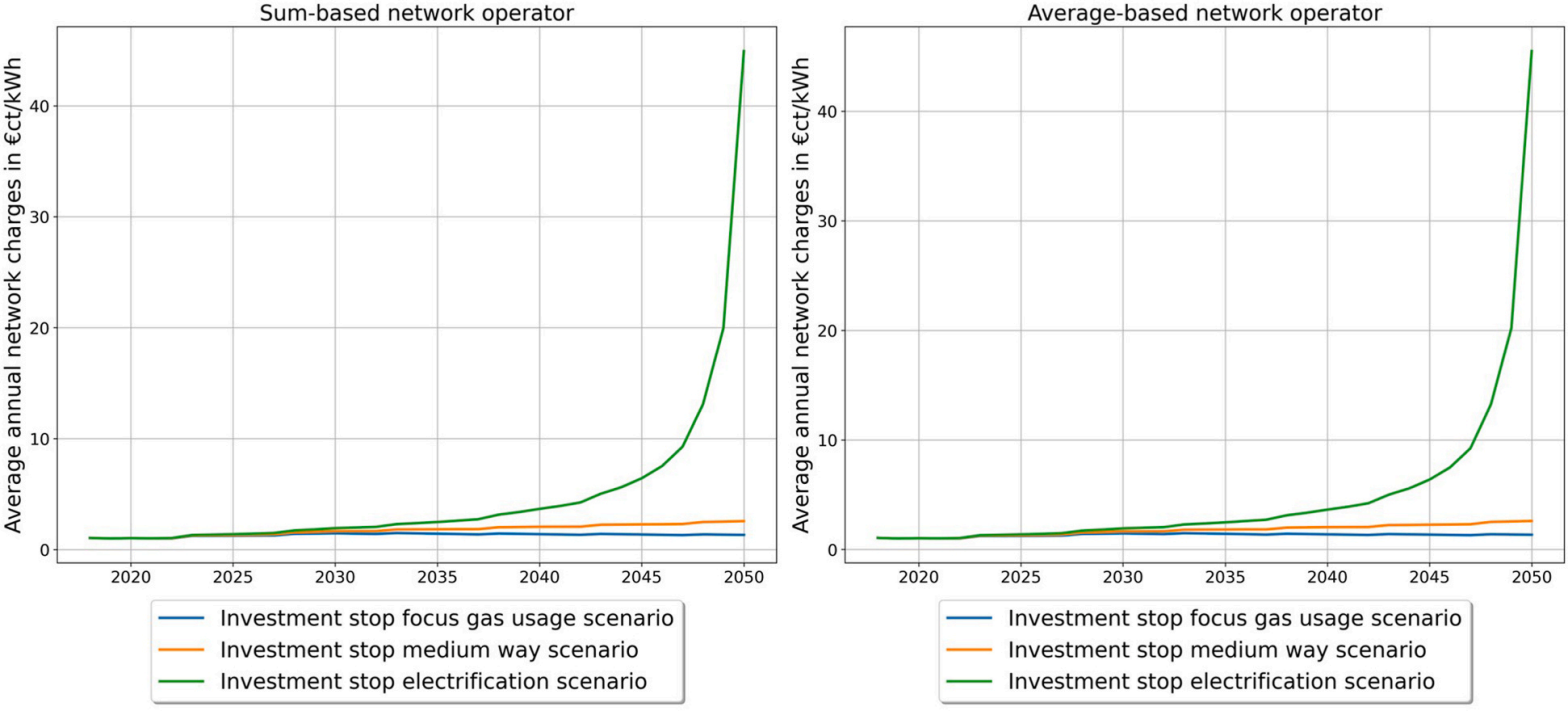
Network charges for the different demand scenarios for the sum-based and average-based network operator (own illustration).

**Table A.10:** NPV for the different investment strategies in the medium way scenarios k = 0.3 and k = 0.9 (own illustration)

Impact on network operator (NPV in thousand € in 2023)	No inclusion of decommission costs in revenue cap	Inclusion of decommission costs in revenue cap	Consideration of provisions
**Investment stop**	−8652		
**Medium way scenario k** = **0.3** **full dismantling**	−29,220	−6998	−15,776
**Medium way scenario k** = **0.3** **partly decommissioning**	−6520	−2710	−6266
**Medium way scenario k** = **0.9** **full dismantling**	−58,229	−2685	−23,693
**Medium way scenario k** = **0.9** **partly decommissioning**	−3269	6253	−3117
**Residual value of the network without reduction of depreciation period**	3495		

**Table A.11:** NPV for the different investment strategies in the gas scenarios k = 0.3 and k = 0.9 (own illustration)

Impact on network operator (NPV in thousand € in 2023)	No inclusion of decommission costs in revenue cap	Inclusion of decommission costs in revenue cap	Consideration of provisions
**Investment stop**	−8652		
**Focus gas usage scenario k** = **0.3** **full dismantling**	−19,275	−6156	−11,614
**Focus gas usage scenario k** = **0.3** partly decommissioning	−7507	−5258	−7588
**Focus gas usage scenario k** = **0.9** **full dismantling**	−37,482	−1719	−16,573
**Focus gas usage scenario k** = **0.9** **partly decommissioning**	−5507	624	−5815
**Residual value of the network without reduction of depreciation period**	3495		

**Fig. A.18:**
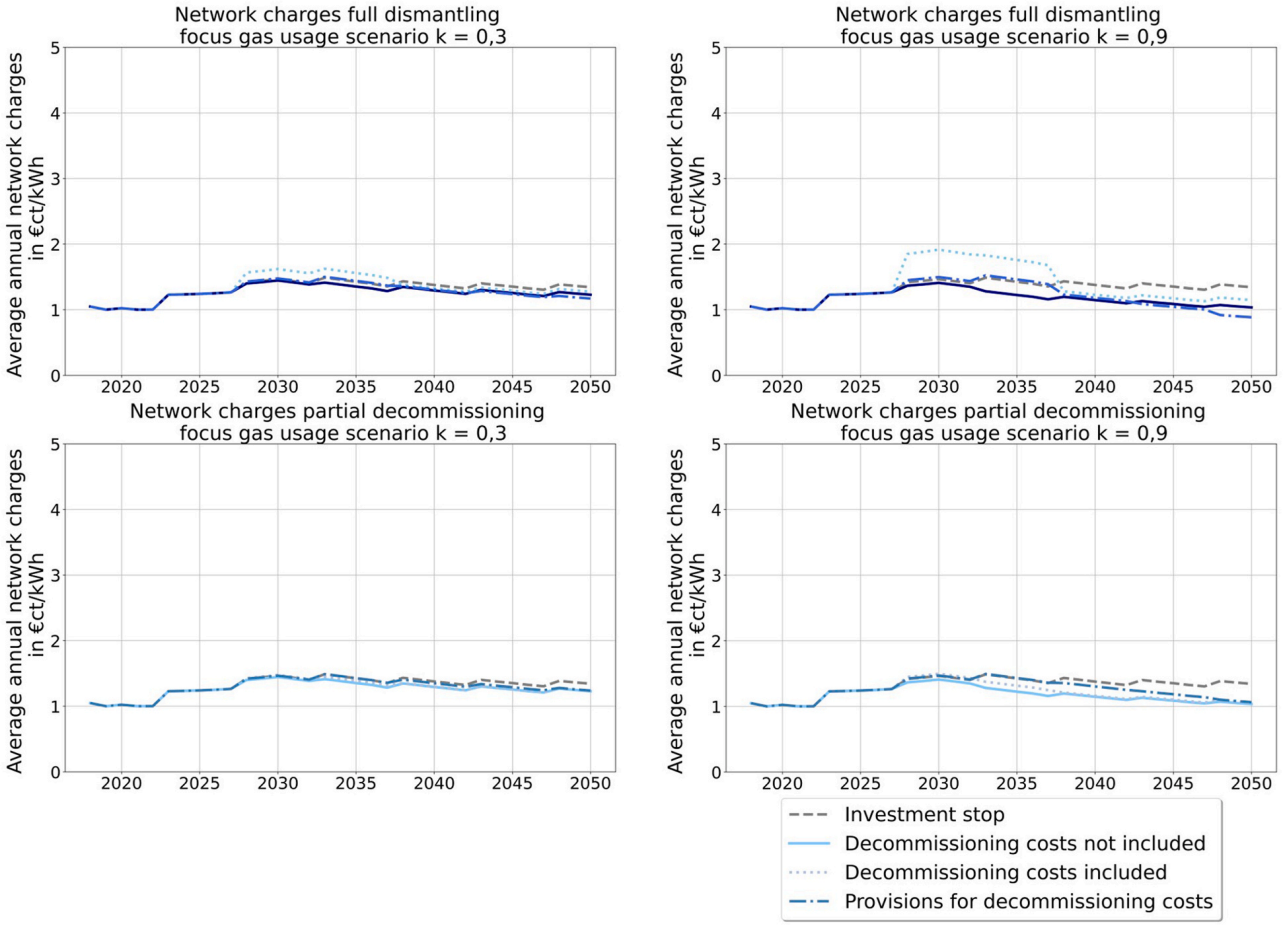
Network charges full dismantling and partial decommissioning of the focus gas usage scenario (own illustration).

**Fig. A.19:**
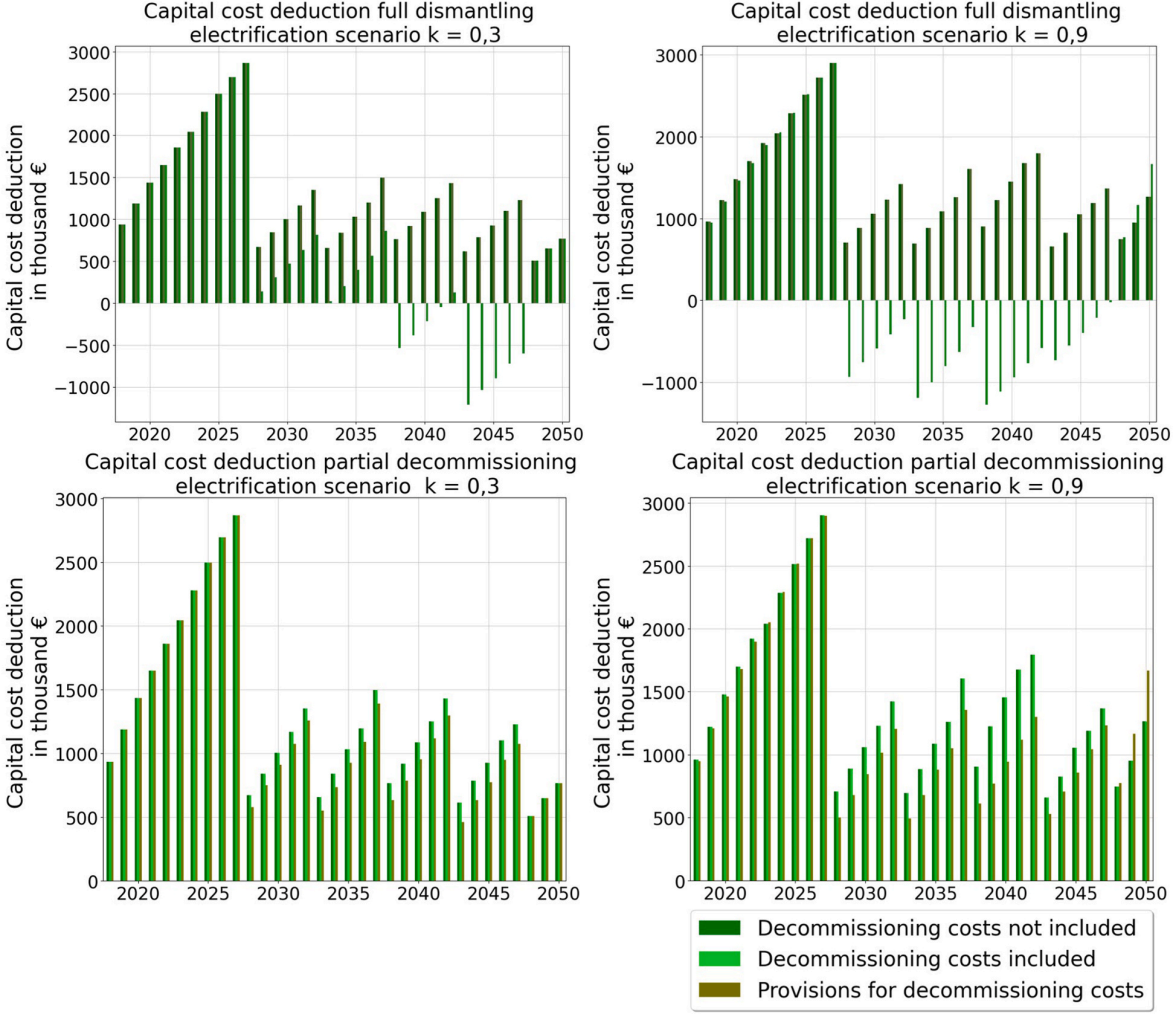
Capital cost deduction for full dismantling and partial decommissioning of the electrification scenario (own illustration).

**Fig. A.20:**
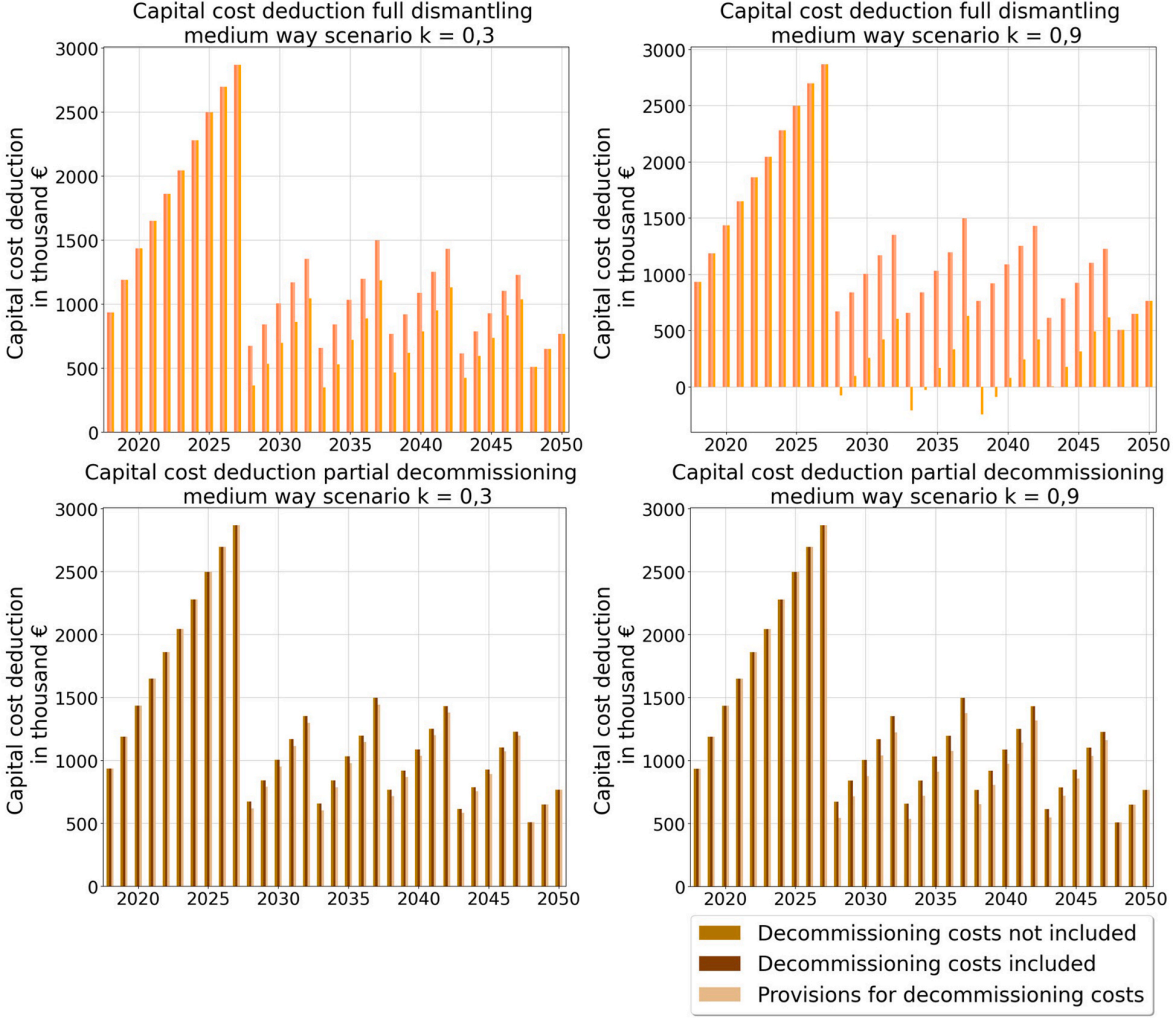
Capital cost deduction for full dismantling and partial decommissioning of the medium way scenario (own illustration).

**Fig. A.21:**
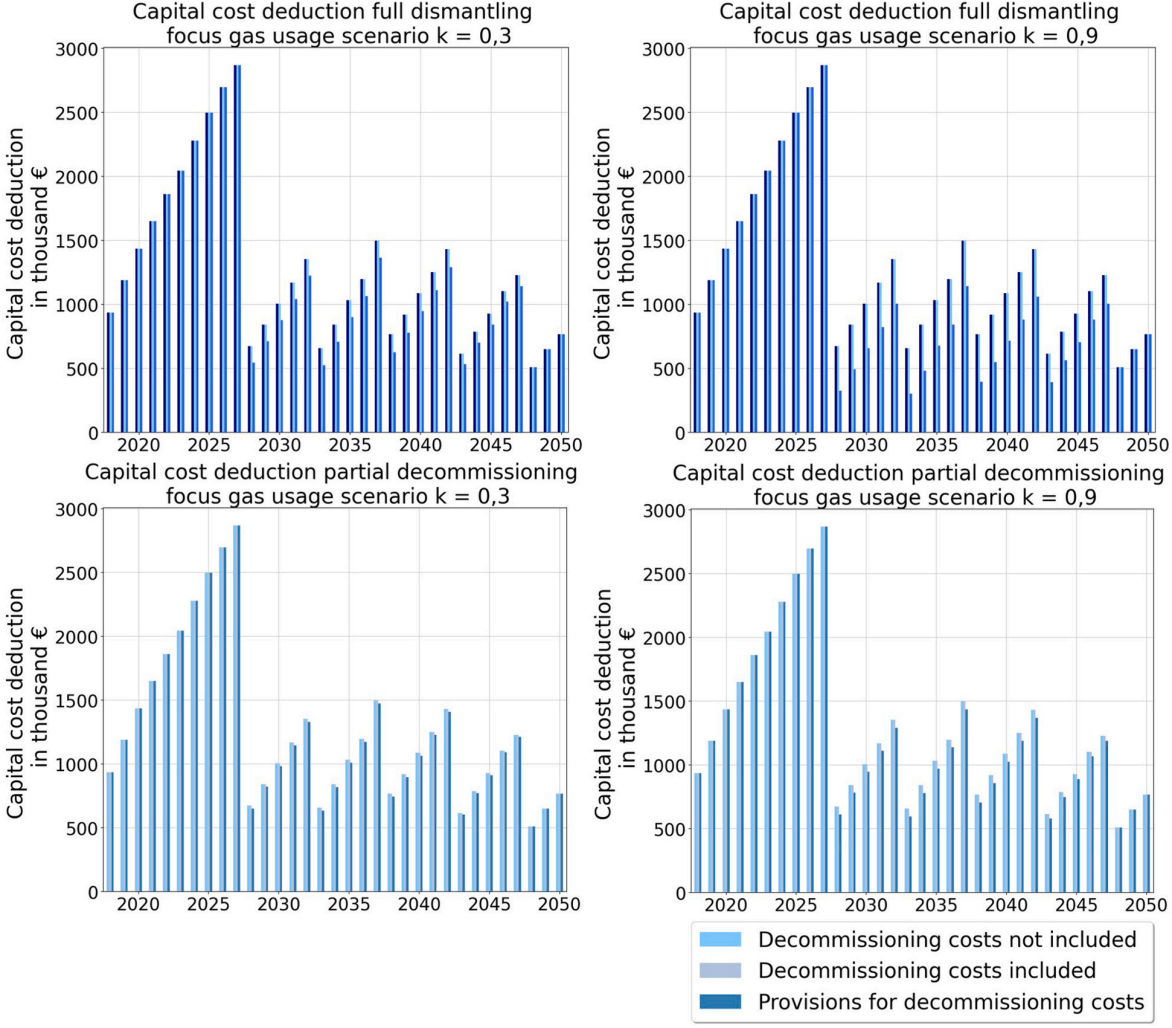
Capital cost deduction for full dismantling and partial decommissioning of the focus gas usage scenario (own illustration).

**Table A.12:** Share of different decommission measures according to real data and literature

	German-wide distribution according to [16]	Distribution based on the data from Karlsruhe	Distribution based on assumptions from DVGW-EBI	German-wide distribution according to [51]	Distribution based on the data from Esslingen
**Dismantling (ND** **> 500** **mm)**	5 %	0.0 %	0 %	0.7 %	0.0 %
**Damming and Sealing (ND 100 mm until 500** **mm**	30 %	62.6 %	38.5 %	48.8 %	61.6 %
**Sealing (DN** < **100** **mm)**	65 %	37.4 %	61.5 %	50.5 %	38.4 %

**Table A.13:** Further assumed shares of different decommission measures

	German-wide distribution according to [16]	No dismantling, low damming	No dismantling, high damming	Medium dismantling	High dismantling
**Dismantling (ND** **> 500** **mm)**	5 %	0 %	0 %	10 %	20 %
**Damming and Sealing (ND 100 mm until 500** **mm**	30 %	5 %	60 %	40 %	40 %
**Sealing (DN** < **100** **mm)**	65 %	95 %	40 %	50 %	40 %

**Fig. A.22:**
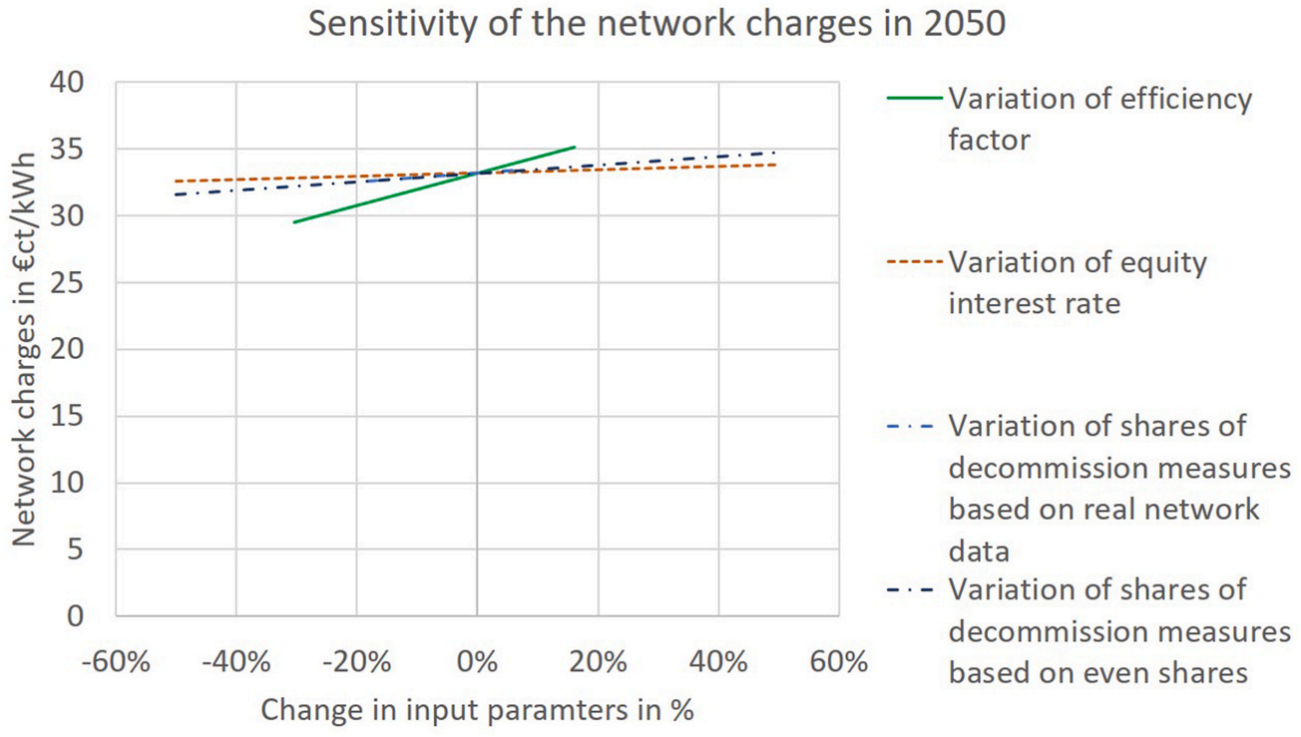
Sensitivity of network charges in 2050 (own illustration).

**Table A.14:** Sensitivity of network charges in 2023 with varying personnel costs (own calculation)

	Proportionate change in personnel costs	Network charges in €ct/kWh
**Personnel costs constant**	233 %	43.3
**Personnel costs increase by 20 %**	300 %	47.1
**Personnel costs decrease to 90 %**	200 %	41.4
**Personnel costs decrease to 60 %**	100 %	35.6
**Personnel costs decrease to 30 % (depending on length)**	0 %	33.2

**Fig. A.23:**
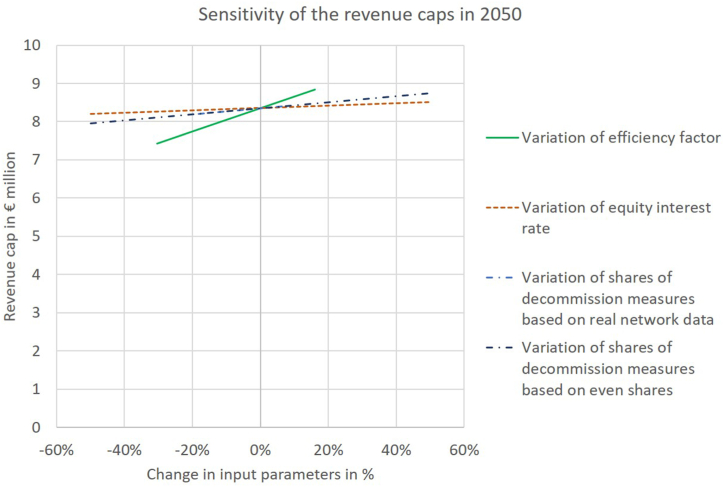
Sensitivity of revenue caps in 2050 (own illustration).

**Table A.15:** Sensitivity of revenue cap in 2050 with varying personnel costs (own calculation)

	Proportionate change in personnel costs	Revenue cap in million €
**Personnel costs constant**	233 %	10.9
**Personnel costs increase by 20 %**	300 %	11.9
**Personnel costs decrease to 90 %**	200 %	10.4
**Personnel costs decrease to 60 %**	100 %	9.0
**Personnel costs decrease to 30 % (depending on length)**	0 %	8.3

## Appendix A

Permanent non-controllable costs (§ 11 ARegV) [31]:

- Legal purchase and payment obligations
- Concession fees
- Operating taxes
- Utilization of upstream network levels
- Works and staff council activities
- Certain approved investment measures of transmission system operators, as well as for electricity distribution system operators (§ 23 ARegV)
- Extended balance equalization § 23 Gas Network Access Directive, minus a rate paid by biogas feeders
- Company and collective agreements on supplementary wage and pension benefits
- Apprenticeship and further training in the company, company day-care centers
- Decisions on cross-border cost sharing
- Research and development according to § 25a ARegV
- Dissolution of network connection cost contributions and building cost subsidies
- Nationwide equalization mechanism according to § 2 par. 5 Energy Grid Expansion Act
- Regulations of the capacity reserve according to § 13e par. 3 EnWG

## Appendix B

The calculated depreciation (${DP}_{t}$) is carried out on a linear basis and considers longer lifetimes, according to annex 1 GasNEV [13], as in commercial law [25]. The assets are differentiated in new and old assets, meaning assets purchased before 2006 are old assets and in as well as after 2006 are new assets. For the calculation of the calculated return on equity, first the equity capital required for operation is determined as the sum of [13]:

• Calculated residual values for old and new assets
• Circulating assets required for operations
• Deduction of the deduction capital
• Borrowed capital (dept)

Besides non-interest-bearing trade payables and interest-free liabilities the deduction capital includes provisions [13].

After determining the equity capital required for operation, it is split into old and new asset shares, each of which is calculated on the basis of total assets [13]. The new asset shares are multiplied by the new asset interest rate and the old asset share by the old asset interest rate, which is slightly lower than the new asset interest rate. These interest rates are set by the regulatory authority before the beginning of the regulation period and the sum of new asset shares and old asset shares result in the return on equity. Based in the return on equity the calculated trade tax is determined by multiplying the return on equity with the trade tax rate and the corresponding assessment rate of the municipality [13]. Lastly, the borrowed capital is included in accordance with its real amount.

In contrast to the capital cost deduction, the capital cost mark-up (${CCM}_{t}$) is determined after the base year during the regulation period, to include new investments taken after the base year in the revenue cap directly and not with a delay in the next regulation period [49]. It corresponds to the capital costs of the new investments, and hence equation (5) is used to determine the capital cost mark-up. While the calculated depreciation, the calculated trade tax and the borrowed capital is determined the same as before, for the return on equity the interest base is the sum of residual value of the new investments minus the residual values for contributions to network connection costs and construction cost subsidies [31]. The interest rate is a weighted mixed interest rate out of the interest rate on equity and the interest rate on borrowed capital for new assets, which both are set by the regulatory authority, as mentioned before.

The efficiency bonus is a bonus given on top of the revenue cap to reward efficient network operators to motivate a continuous efficient network operation even after achieving an efficiency factor of 100 % [29]. The consumer price index (CPI) is the same for all network operators and serves to correct inflation [25]. With the assumption that productivity in the network economy increases faster than the overall economic average, the general sectoral productivity factor is set by the regulatory authority and is the same for all network operators. To limit the risk that efficiency is realized by lowering the security standards the quality element was introduced for electricity networks. This is not done so far for the gas network, however research is conducted by Ref. [50] on how this quality element could be determined for gas networks [25]. argue that the technical regulation for gas networks is already very restrictive so that there is little possibility to improve efficiency on cost of security. The volatile costs vary from year to year strongly, such as the loss of energy. Consequently, these costs are not necessarily efficient, but still underly the efficiency comparison [25]. Lastly, with the balance of the regulation account the actual and the approved revenues are recorded and balanced [29].

## Appendix C

For the validation of the model results, the calculated and real values for the revenue cap during the regulation period, in other word including the capital cost mark-up, is shown in Table A7 This table only includes the values until 2020, because the data for the new investments is only available until 2020. The deviations between the real and calculated values show a difference between 0.44 % and 0.08 % for SWKN, whereas these are between 0.34 % and 0.16 % for SWE.

The comparison of the calculated and real temporarily non-controllable costs and controllable costs for SWKN (Table A8) illustrates differences between 0.24 % and 0.16 %.

## Appendix D

Two paths of anonymizing the data are chosen. The first one creates one big network operator by summing up the real data from the different network operators to one. The second way is to build the average of data from the network operators. Table A.9 provides an overview of the two ways and the input data addressed. The lease payments of SWKN are excluded, so that data distortion is prevented, since one real network operator is leasing the network components, while the other one is not. Because of the nature of the input date for the trade tax rate and the efficiency factor only the average results in a useful number, while the sum would be out of range allowed. For the asset portfolio summing up can easily be conducted, while to calculate the average it has to be determined for every asset with the same type, purchase year and life span. The rest of the data, the sum or the average can be determined, whereas the data in the last column of Table A.9 stay the same for all network operators.

The resulting fictitious network operators are compared to the revenue cap of Karlsruhe and Esslingen in Figure A.12 and Figure A.13. Blue shows the resulting revenue cap for the sum-based network operator on the left (Figure A.12) and for the average-based network operator on the right (Figure A.13). The orange bars are the revenue caps for Karlsruhe and the green for Esslingen. It can be seen that the sum-based network operator is closer to Karlsruhe, representing a big network operator, whereas the average-based network operator is closer to Esslingen, showing a smaller network operator.

## Appendix E

The asset portfolio of the average-based network operator and an overview of the initial input data valid for all investment options investigated are included in the two datasets at https://fordatis.fraunhofer.de/handle/fordatis/334 (Oberle, S.; Gnann, T.; Wayas, L; Wietschel, M., 2023). This is an online data repository hosted at Fordatis - Research DataRepository of Fraunhofer-Gesellschaft.

## Appendix F

For the investment stop strategy, the residual values of the sum-based (left side of Figure A.14) and average-based (right side of Figure A.14) network operator are investigated. It can be seen that after the third regulation period (2018–2022) the residual values jump up, because between the base years 2015 and 2020 new investments were made, leading to an increased network value. After 2020 no new investments are made, leading to a reduction of the network value in the next decades. However, in 2050 residual values of more than 20,000 thousand € occur for the sum-based network operator and more than 10,000 thousand € for the average-based network operator.

Based on the residual values the capital costs are calculated and the results are illustrated in Figure A.15. Again, on the right side the sum-based network operator is shown and on the left the average-based one. It can be seen that for both network operators the depreciation is the highest share of capital costs, followed by the return on equity, which decreases slightly faster for the sum-based network operator than for the average-based one. This is due to the different asset bases. Overall, the capital costs decrease similar to the residual values.

In contrast, the revenue cap of both networks only decreases slightly, even though no new investments are taken after 2020, as shown in Figure A.16. This is due to the high share of OPEX, such as material costs or personnel costs, because even though we do not see new investments in this investment option the networks are still operated, leading to continues OPEX. Nonetheless, the development of revenue cap is nearly identical for the sum-based and average-based network operator, with only higher values for the sum-based one.

Lastly, with the network charges the impact of the investment option and the demand development on the network user can be analyzed. Figure A.17 shows the development of the network charges in the different demand scenarios for the sum-based (left side) and average-based (right side) network operator. For both network operators the charges are almost the same, due to the same development of investments and demand assumed. Further, it can be seen that for the focus gas usage scenario the network charges stay nearly the same, and for the medium way scenario only a slight increase in network charges appears. In contrast, in the electrification scenario the network charges experience an exponential growth up to around 45 €ct/kWh.

## Appendix G

In the medium way scenario with k = 0.3, all the options result in negative NPV, hence losses for the network operator (Table A.10). Nonetheless, the inclusion of the decommissioning costs results in the lowest losses for partial decommissioning and full dismantling. While the consideration of provisions leads to similar losses as the non-inclusion of decommissioning costs for the partial decommissioning, for the full dismantling it is preferable for the network operator compared to not including the decommissioning costs into the revenue cap. In this scenario all partial decommissioning options are preferable compared to the investment stop, and even the inclusion of full dismantling costs leads to lower losses.

Similar results as for the electrification scenario k = 0.9 are achieved in the medium way scenario k = 0.9, shown in the lower two rows of Table A.10. Only the inclusion of partial decommissioning costs in the revenue cap results in a positive NPV, whereas the other two options for the partial decommissioning are on a similar level. Further, including the cost of full dismantling in the revenue cap results in slightly lower losses than the two options previously mentioned and these options are preferable over doing nothing as the comparison with the NPV of the investment stop shows.

The focus gas usage scenarios result in similar developments as the medium way scenarios, as Table A.11 shows.

## Appendix H

Similar to the medium way scenarios, the network charges for the focus gas usage scenarios vary less than 1 €ct/kWh. A slightly higher variation of network charges shows the focus gas usage scenario k = 0.9 with full dismantling compared to the other focus gas usage scenarios. It clearly depicts the increase of network charges due to inclusion of full dismantling costs in the revenue cap (dotted line) and further shows the provision effect of first higher and later lower network charges (dash-dotted line).

## Appendix I

As mentioned in section 4.2.4 the consideration of provisions leads to a reduction in capital cost deduction. Nonetheless, a negative capital cost deduction is a breach of the current regulation. Figure A.19 compares the capital cost deduction for the electrification scenarios. While the electrification scenarios with partial decommissioning show only a slight decrease in capital cost deduction, the electrification scenarios with full dismantling result into negative capital cost deductions not allowed by the current regulation.

A similar breach in regulation appears for the medium way scenario k = 0.9 full dismantling (Figure A.20 upper right), while the other medium way scenarios only show a slight reduction in capital cost deduction, as intended by the regulation.

For the focus gas usage scenarios, no breach of regulation appears as Figure A.21 shows.

## Appendix J

Table A.12 provides an overview of the different shares of decommission measures based on real networks for Karlsruhe and Esslingen, as well as for statistical data such as the German-wide share of the different pipeline diameters considered.

Further, to expand the variation of decommission measure shares more even distributions of the measures are assumed, as illustrated in Table A.13.

## Appendix K

Additional to the sensitivity of the NPV, explained in section 4.3, the effect of the variations on the network charges in 2050 (Figure A.22) and on the revenue cap in 2050 (Figure A.23) is analyzed. In Figure A.22 the variation in equity interest rate (orange dashed line) has only a slight effect on the network charges, as well as the changing shares of decommission measures. Solely the efficiency factor varies the network charges between less than 30 €ct/kWh and slightly more than 35 €ct/kWh.

Table A.14 provides an overview of the effect of personnel cost changes on the network charges, leading to the conclusion the higher the costs the higher the network charges.

Similar to the network charges the parameter affecting the revenue cap most is the efficiency factor, while variation of equity interest rate and different shares of decommission measures only has a slight effect of around one million € (Figure A.23).

Also, the increase in personnel costs leads to an increase in revenue cap (Table A.15), as shown already for the network charges.

## Footnotes

1 This paper addresses still the target of climate-neutrality until 2050 for Germany. Nonetheless, this work provides valuable insights into the interdependencies between regulation, network operator and user. Further the insights gained and conclusions drawn are valid for an energy system reaching climate neutrality until 2045. The resulting costs will most likely increase because of the faster transition.

2 Appendix A provides an overview of the costs counting as permanent non-controllable costs according to § 11 ARegV [31].

3 The updated version of the scenarios chosen in this paper (explained in Section 3.2.1) were published in November 2022, so that a consideration in this work was timewise not possible. Nonetheless, the insights gained for the target of climate neutrality in 2050 are valuable for the new target as well, which mainly will speed up the transition processes or increase the costs.
